# Health-Promoting Lifestyle Scores, Academic Stress, and Health-Professional Advice Seeking Among Undergraduate Nursing Students: A Cross-Sectional Study

**DOI:** 10.3390/ejihpe16070101

**Published:** 2026-07-15

**Authors:** Alexis Emmanuel Salinas-Santoyo, Gabriela Luna-Hernández, Victor Horacio Orozco-Covarrubias, Janvier Andre Martinez-Godinez, Jaime Briseno-Ramírez, Cecilia Alejandra Zamora-Figueroa

**Affiliations:** 1 Departamento de Enfermería para la Atención, Desarrollo y Preservación de la Salud Comunitaria, Especialidad de Enfermería en Salud Pública, Centro Universitario de Ciencias de la Salud, Universidad de Guadalajara, Guadalajara 44340, Jalisco, Mexico; alexis.salinas2212@alumnos.udg.mx (A.E.S.-S.); janvier.martinez@alumnos.udg.mx (J.A.M.-G.); 2 Departamento de Alimentación y Nutrición, Centro Universitario de Ciencias de la Salud, Universidad de Guadalajara, Guadalajara 44340, Jalisco, Mexico; gabriela.lhernandez@academicos.udg.mx; 3 Departamento de Salud-Enfermedad como Proceso Individual y Colectivo, Centro Universitario de Tlajomulco, Universidad de Guadalajara, Tlajomulco de Zuniga 45641, Jalisco, Mexico; horacio.orozco@academicos.udg.mx; 4 División Salud, Centro Universitario de Tlajomulco, Universidad de Guadalajara, Tlajomulco de Zuniga 45641, Jalisco, Mexico; 5 Hospital Civil de Oriente, Tonala 45425, Jalisco, Mexico

**Keywords:** health-promoting lifestyle, HPLP-II, nursing students, academic stress, health-professional advice, propensity score, IPTW, robust regression

## Abstract

Undergraduate nursing students are trained to promote health in clinical and community settings, but their own health-promoting behaviors occur in the context of academic demands, clinical training, work responsibilities, and limited time for self-care. The primary objective of this cross-sectional analytic study was to estimate the adjusted association between health-professional advice seeking and global Health-Promoting Lifestyle Profile II (HPLP-II) scores among 506 undergraduate nursing students at the Centro Universitario de Ciencias de la Salud, Universidad de Guadalajara. Secondary analyses described HPLP-II scores, stress ratings, information sources, subscale-specific associations, and propensity-score sensitivity analyses; exploratory analyses evaluated HPLP-II psychometrics, level-based lifestyle profiles, and stress-by-advice-seeking interaction. The overall median HPLP-II score was 2.40 (IQR: 2.06, 2.79). Internal consistency was high for the global scale (Cronbach’s alpha = 0.961) and ranged from acceptable to high across subscales (alpha = 0.812–0.900). Ordinal exploratory factor analysis using polychoric correlations supported exploratory use of the theoretical six-domain structure but did not provide confirmatory validation; parallel analysis suggested eight factors. Two level-based lifestyle profiles were identified: Low HPLP (58.1%) and High HPLP (41.9%), reflecting broad score-level separation rather than distinct validated phenotypes. In the primary HC3 robust model, health-professional advice seeking was associated with higher global HPLP-II scores (*b* = 0.242, 95% CI: 0.140, 0.344; *p* < 0.001), whereas academic stress and vacation-period stress showed small inverse adjusted associations with HPLP-II scores. Sensitivity analyses, including IPTW, a modified HPLP-II score excluding Health Responsibility, and a model excluding willingness to improve lifestyle, showed advice-seeking coefficients in the same positive direction. The exploratory stress-by-advice-seeking interaction was not statistically significant. Findings should be interpreted as associations rather than causal effects.

## 1. Introduction

Undergraduate education overlaps with a developmental period in which many young adults negotiate new autonomy, social roles, health behaviors, and professional identity (Arnett, 2000). In nursing, this transition occurs within a professional program that combines theoretical coursework, examinations, clinical rotations, and early responsibility for patient-centered care (Chernomas & Shapiro, 2013; Labrague, 2024; Pulido-Martos et al., 2012). These demands frequently coexist with irregular schedules, sleep disruption, financial strain, employment, and concern about clinical performance, factors repeatedly linked to stress, psychological distress, and inconsistent self-care among nursing students (Li & Hasson, 2020; Pulido-Martos et al., 2012; Vo et al., 2023).

Health-promoting lifestyles refer to self-initiated behaviors intended to maintain or improve well-being and are central to Pender’s health promotion model (Kuan et al., 2019; Pender et al., 2015; Walker et al., 1987). The Health-Promoting Lifestyle Profile II (HPLP-II) operationalizes this construct through six dimensions: Health Responsibility, Nutrition, Physical Activity, Stress Management, Spiritual Growth, and Interpersonal Relations (Pérez-Fortis et al., 2012; Walker et al., 1987; Zambrano Bermeo et al., 2024). Nursing students are an important population for this construct because they are preparing to promote preventive behaviors while developing personal habits that are relevant to future professional role modeling (Hwang & Oh, 2020; Mak et al., 2018; Stanulewicz et al., 2020).

International studies indicate that nursing students do not uniformly translate health-related knowledge into health-promoting lifestyle practices (Al-Kandari et al., 2008; Chow et al., 2018; Gurusamy et al., 2022; Mak et al., 2018). Physical activity, nutrition, rest, and stress-management behaviors are commonly reported as weaker areas, suggesting that health-related knowledge may coexist with less consistent health-promoting behavior under demanding educational conditions (Gawlik et al., 2024; Hwang & Oh, 2020; Slemon et al., 2021). This pattern is relevant for nursing education because self-care is often discussed in relation to resilience, well-being, and the capacity to model health-promoting practices in clinical and community settings (Slemon et al., 2021; Stanulewicz et al., 2020).

Evidence from Spanish-language and Latin American university samples also indicates that HPLP-II applications require local psychometric checking rather than assuming that dimensional findings transport unchanged across educational and cultural contexts (Enríquez Reyna et al., 2022; Pérez-Fortis et al., 2012; Zambrano Bermeo et al., 2024). Mexican evidence remains limited for undergraduate nursing students, particularly for studies that jointly examine health-promoting lifestyle scores, academic stress, and health-professional advice seeking using transparent multivariable and sensitivity analyses.

Academic stress is one of the most consistent contextual challenges reported in nursing education (Labrague, 2024; Pulido-Martos et al., 2012; Vo et al., 2023). Systematic reviews and empirical studies link nursing-student stress to psychological well-being, coping, burnout, and academic or clinical performance concerns (Chernomas & Shapiro, 2013; Gibbons et al., 2010; Li & Hasson, 2020). These findings support examining stress not only as an outcome of the educational environment but also as a potential correlate of health-promoting lifestyle behaviors (Berdida & Grande, 2022; Hwang & Oh, 2020; Pulido-Martos et al., 2012).

Health-professional advice seeking and other forms of help-seeking may reflect or involve access to nutrition, psychological, medical, rehabilitation, or physical-activity guidance, but help-seeking is a staged and socially patterned behavior rather than a randomly assigned exposure (Alqhtani et al., 2025; Bamine & Tanaka, 2025). Students who seek professional advice may differ from non-seekers in perceived need, motivation, health awareness, stigma, service availability, and access to resources, which makes crude comparisons vulnerable to self-selection and confounding (Alqhtani et al., 2025; Austin, 2011; Bamine & Tanaka, 2025). Propensity score methods, including inverse probability of treatment weighting, can improve balance on observed covariates in observational studies, although they do not remove unmeasured confounding or justify causal interpretation in cross-sectional designs (Austin, 2011; Austin & Stuart, 2015; Rosenbaum & Rubin, 1983).

The primary research question was whether health-professional advice seeking was associated with global HPLP-II scores after adjustment for sociodemographic, academic, behavioral, household, and health-information covariates. Secondary objectives were to describe HPLP-II scores and estimate subscale-specific, empirical lowest-quartile, and IPTW sensitivity analyses. Exploratory objectives were to evaluate internal consistency and ordinal exploratory dimensional evidence for the HPLP-II, describe level-based lifestyle profiles, and test whether the stress-HPLP-II association differed by health-professional advice seeking (Austin, 2011; Enríquez Reyna et al., 2022; McClelland & Judd, 1993; Pérez-Fortis et al., 2012; Zambrano Bermeo et al., 2024). Because the study is cross-sectional, all inferential language is framed in terms of association rather than causation (Rosenbaum & Rubin, 1983; von Elm et al., 2007).

## 2. Materials and Methods

### 2.1. Study Design and Setting

A cross-sectional observational study was conducted during the 2025 academic period at the Centro Universitario de Ciencias de la Salud (CUCS), Universidad de Guadalajara, Jalisco, Mexico, and the reporting structure followed STROBE recommendations for observational studies (von Elm et al., 2007). The source workbook retained valid questionnaire timestamps from 13 May 2025 to 20 May 2025. The target population comprised undergraduate students enrolled in the Bachelor of Science in Nursing program.

### 2.2. Participants and Sampling

The required sample size was calculated using OpenEpi version 3.01 for a finite population of 2455 students, a 97% confidence level, and a 5% margin of error, yielding a minimum sample of 396 participants. The 97 percent level was set a priori as a slightly more conservative precision target than the conventional 95 percent level, which marginally increased the minimum sample required for the descriptive estimate. The original confidence level was used only for the descriptive precision calculation; all model estimates are reported with 95% confidence intervals. This precision-based estimate assumed maximum variability for a descriptive proportion and was not intended as a formal power calculation for multivariable, factor-analytic, weighting, or interaction analyses. To increase precision, 506 complete analytic records were included. A semester-stratified recruitment strategy was used, with academic semester serving as the stratification variable. Students were approached through the electronic questionnaire workflow used by the academic program, and participation was voluntary, anonymous at the analytic stage, and not linked to course credit or grading. Because participation was voluntary through an electronic form and the de-identified analytic file did not retain invitation-tracking information, the number invited by stratum and the response rate could not be reconstructed; representativeness is therefore interpreted cautiously.

### 2.3. Data Collection

Data were collected electronically using Google Forms. The questionnaire included sociodemographic variables, behavioral variables, perceived academic stress, health-professional advice seeking, willingness to improve lifestyle, and the HPLP-II. Participation was voluntary. All 506 analytic participants signed the informed-consent form before completing the questionnaire, and no directly identifying personal variables were retained in the analytic dataset.

### 2.4. Measures

#### 2.4.1. Sociodemographic and Behavioral Variables

Students reported age, sex, current semester, employment status, weekly working hours, financial support, economic-support source, current residence, housing type, household composition, tobacco smoking, alcohol consumption, and main source of health information. Employment status was coded as study only or study and work. Weekly working hours were grouped as no work, flexible shift (<24 h), part-time (24 h), and full-time (48 h). Economic-support source was grouped as none, parents, scholarship, or partner/family. Residence categories were Guadalajara, Zapopan, San Pedro Tlaquepaque, Tonalá, and other municipalities. Housing type was coded as owned, rented, or borrowed; household composition as family, alone, friends, or partner; and health-information source as social media, official websites, research articles, books, or other sources. Binary tobacco and alcohol indicators were used in the outcome model; ordinal frequency scores from the original response options were used in the propensity-score model.

#### 2.4.2. Health-Professional Advice Seeking

Health-professional advice seeking was measured with the original Spanish item, “¿Acudes con un profesional para asesoría en temas de salud, nutrición, manejo del estrés o actividad física?” This was translated as “Do you consult a professional for advice on health, nutrition, stress management, or physical activity?” Response options were Yes and No, and no explicit recall or reference period was specified. Health-professional advice seeking was coded as a binary variable indicating whether the student reported advice seeking from health-related professionals, including physicians, nutritionists, psychologists, therapists, or physical trainers. This variable was conceptualized as a support-seeking behavior and not as a randomized intervention because help-seeking among students in the health professions depends on perceived need, attitudes, stigma, disclosure, and access to services (Alqhtani et al., 2025; Bamine & Tanaka, 2025). The questionnaire did not record the specific type of professional consulted, so this measure represents broad health-professional support seeking rather than a specific advice or support modality. This heterogeneity may average domain-specific pathways, such as nutrition-oriented advice being more closely related to Nutrition scores and psychological support being more closely related to Stress Management scores.

#### 2.4.3. Perceived Academic Stress and Willingness to Improve

Students rated perceived stress during active academic periods and vacation periods from 0 (no stress) to 10 (extreme stress). The vacation-period stress item used the same anchors and was included to distinguish stress during academic activity from stress during lower-demand periods. Willingness to improve lifestyle was self-rated from 0 (lowest willingness) to 10 (highest willingness). These single-item numerical ratings were used to reduce response burden in a questionnaire that also included the 52-item HPLP-II; they were analyzed as perceived ratings rather than as multidimensional psychometric scales. They should therefore be interpreted as brief self-appraisals and not as substitutes for validated multi-item stress or motivation instruments.

#### 2.4.4. Health-Promoting Lifestyle Profile II

Health-promoting lifestyles were measured using the Spanish-translated version of the Health-Promoting Lifestyle Profile II (HPLP-II), based on Pender’s health promotion model (Pender et al., 2015; Walker et al., 1987, 1995). The instrument contains 52 items scored on a 4-point Likert scale from 1 (Never) to 4 (Routinely), and it includes six dimensions: Health Responsibility, Nutrition, Physical Activity, Stress Management, Spiritual Growth, and Interpersonal Relations (Kuan et al., 2019; Pérez-Fortis et al., 2012; Walker et al., 1987). Global and subscale scores were calculated as item means, yielding a range from 1.00 to 4.00 (Al-Kandari et al., 2008; Zambrano Bermeo et al., 2024). Because Spanish-language applications of the HPLP-II may vary across student populations, internal consistency, corrected item-total correlations, factorability, and exploratory dimensional structure were evaluated in this sample (Enríquez Reyna et al., 2022; Pérez-Fortis et al., 2012; Zambrano Bermeo et al., 2024).

### 2.5. Statistical Analysis

Categorical variables were summarized as frequencies and percentages. Continuous variables were examined using Shapiro–Wilk tests and visual diagnostics (Shapiro & Wilk, 1965). Because age and HPLP-II scores were non-normally distributed, they were summarized using medians and interquartile ranges (IQR). Sex-based comparisons used Chi-square tests or Fisher’s exact tests for categorical variables and Wilcoxon rank-sum tests for continuous variables. Statistical significance was set at p<0.05.

Psychometric analyses included Cronbach’s alpha, standardized alpha, corrected item-total correlations, Kaiser–Meyer–Olkin (KMO) factorability diagnostics, and Bartlett’s test of sphericity (Bartlett, 1950; Cronbach, 1951; Kaiser & Rice, 1974). Because HPLP-II items use four ordered response categories, exploratory ordinal dimensional analyses were added using polychoric correlations, minimum-residual extraction, and oblimin rotation. Parallel analysis with polychoric correlations was used to examine dimensionality. A six-factor ordinal EFA was retained for descriptive comparison with the theoretical HPLP-II domains, not as confirmatory validation. Principal component analysis (PCA) was used only as a descriptive eigenvalue check. Lifestyle profiles were identified using k-means clustering on the six HPLP-II subscale scores (Lloyd, 1982). Before clustering, subscale scores were z-standardized to reduce the influence of differences in subscale variability. Candidate solutions from two to five clusters were fitted with 100 random starts and compared using average silhouette width; the final number of clusters was selected primarily by the largest average silhouette value, with interpretability of the resulting profiles considered secondarily. Profiles were interpreted as level-based lifestyle profiles because the selected solution primarily separated lower versus higher scores across all six domains. To assess whether the clustering added information beyond a simple global-score split and how much it depended on the theoretical subscale structure, the two-profile solution was compared with a median split of the global HPLP-II score (agreement and Cohen’s kappa) and with a k-means solution computed on principal-component scores derived from the 52 items instead of the theoretical subscales. The selected solution was described using cluster centers, profile frequencies, and associations with health-professional advice seeking, information source, stress, and willingness to improve lifestyle (Rousseeuw, 1987).

The primary outcome was the global HPLP-II score. The primary analysis was the multivariable HC3 robust linear model for global HPLP-II score. The primary multivariable model included health-professional advice seeking, the single-item perceived academic stress rating during the academic period, the single-item vacation-period stress rating, willingness to improve lifestyle, age, sex, semester, working hours, economic support, residence, housing type, household composition, health-information source, tobacco use, and alcohol use. Reference categories for all categorical covariates are reported with the primary model results. The complete model including every covariate is provided in Supplementary Table S10. Covariates were selected a priori from the questionnaire domains because they represented sociodemographic, academic, behavioral, household, and information-source factors plausibly associated with both advice seeking and lifestyle scores. Heteroskedasticity-consistent HC3 robust standard errors were used for inference (Hayes & Cai, 2007; MacKinnon & White, 1985). Coefficients are unstandardized and interpreted as adjusted mean differences in HPLP-II scores associated with each covariate. Because the study specified a single primary hypothesis—the adjusted association between health-professional advice seeking and global HPLP-II score—the primary model was treated as confirmatory for that one association and was not subjected to multiple-comparison correction, while the remaining primary-model terms were interpreted as adjustment covariates rather than as independent confirmatory tests. The subscale-specific, secondary lowest-quartile, propensity-score, additional sensitivity, and exploratory interaction models were regarded as supportive or hypothesis-generating analyses of this same primary association rather than as a separate family of confirmatory hypotheses. Secondary analyses included subscale-specific models and an empirical lowest-quartile HPLP-II logistic model. Exploratory analyses included psychometrics, level-based profiles, and the stress-by-advice-seeking interaction. Subscale-specific models used the same covariate structure, with Benjamini–Hochberg false-discovery-rate adjustment across key terms (Benjamini & Hochberg, 1995). Two additional HC3 sensitivity models were conducted: one using a modified global HPLP-II score excluding the Health Responsibility subscale to address conceptual overlap with health-professional advice seeking, and one refitting the primary model without willingness to improve lifestyle because its temporal role could not be established in the cross-sectional design.

To evaluate the robustness of the health-professional advice association, a propensity score for health-professional advice seeking was estimated using observed covariates and applied through stabilized inverse probability of treatment weighting (IPTW) (Austin, 2011; Austin & Stuart, 2015; Rosenbaum & Rubin, 1983). The propensity-score model included age, sex, semester, working-hours category, economic-support source, residence, housing type, household composition, health-information source, academic-period stress, vacation-period stress, willingness to improve lifestyle, tobacco frequency, and alcohol frequency. Weights were truncated at the 1st and 99th percentiles, and covariate balance was evaluated using standardized mean differences (Austin, 2009; Austin & Stuart, 2015). Weighted outcome models used HC3 robust sandwich standard errors to account for heteroskedasticity under weighting. Because including an estimated propensity score directly as a regression covariate can create bias under some conditions, IPTW was treated as the primary propensity-score sensitivity analysis rather than as the main outcome model (Hade & Lu, 2013). Complete-case analysis was used for multivariable models; after recoding, all 506 analytic records had complete data for the primary model. A secondary logistic model examined empirical lowest-quartile HPLP-II status, defined as scores at or below the sample 25th percentile; this threshold was not treated as a validated clinical category. An interaction term between academic stress and health-professional advice seeking was added in a separate exploratory model. Because interaction tests often have lower statistical power and because this analysis was not the primary endpoint, the moderation results are interpreted cautiously (McClelland & Judd, 1993).

### 2.6. Software

All analyses were conducted in R version 4.5.3 (11 March 2026). The R packages used in the reproducible pipeline were readxl 1.4.5, dplyr 1.2.1, tidyr 1.3.2, ggplot2 4.0.2, broom 1.0.12, sandwich 3.1-1, lmtest 0.9-40, cobalt 4.6.2, openxlsx 4.2.8.1, purrr 1.2.1, stringr 1.6.0, forcats 1.0.1, cluster 2.1.8.2, knitr 1.51, scales 1.4.0, and ggsci 4.2.0. The de-identified analytic dataset, R scripts, documentation, and reproducibility files are archived in Zenodo at https://doi.org/10.5281/zenodo.20382383.

## 3. Results

### 3.1. Sample Characteristics

The final sample included 506 undergraduate nursing students. Most participants were female (79.6%), reflecting the gender distribution commonly observed in nursing programs. The median age was 21.0 years (IQR: 20.0, 22.8), with no statistically significant sex-based difference in age (p=0.067).

Employment status differed by sex (p=0.030), with a higher proportion of male students combining study and work compared with female students (58.3% vs. 45.7%). Weekly working hours also differed by sex (p=0.012), with full-time work reported by 21.4% of male students and 10.4% of female students. Financial support was more common among female students than male students (80.4% vs. 64.1%, p<0.001).

Current residence, health-professional advice seeking, tobacco smoking, and alcohol consumption did not differ significantly by sex. Overall, 26.7% of students reported seeking health-professional advice, 8.7% reported tobacco smoking, and 62.8% reported alcohol consumption. Full descriptive results for Table 1 are shown below, and a complete summary of all covariates used in the multivariable and propensity-score models is provided in Supplementary Table S1.

### 3.2. HPLP-II Scores

**Figure 1:**
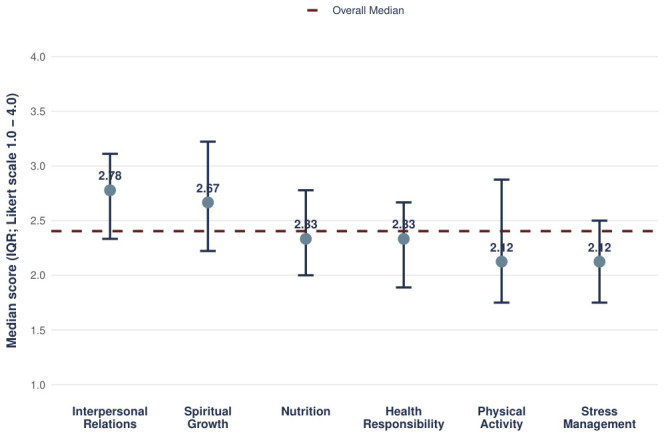
HPLP-II dimension scores of undergraduate nursing students. (N=506). Points show subscale medians and error bars show interquartile ranges. The dashed line represents the overall HPLP-II median as a visual reference.

The overall median HPLP-II score was 2.40 (IQR: 2.06, 2.79) on the 1.00–4.00 scale. The highest median subscale scores were observed for Interpersonal Relations (2.78; IQR: 2.33, 3.11) and Spiritual Growth (2.67; IQR: 2.22, 3.22). The lowest median scores were observed for Stress Management (2.12; IQR: 1.75, 2.50) and Physical Activity (2.12; IQR: 1.75, 2.88). Consistent with the non-normal HPLP-II score distributions, Figure 1 displays subscale medians and interquartile ranges, and the dashed reference line uses the overall HPLP-II median.

Global HPLP-II scores did not differ significantly by sex (p=0.448). Most subscales also showed no statistically significant sex-based differences. Physical Activity was the exception, with male students reporting higher scores than female students (median 2.50 vs. 2.00; p<0.001). The HPLP-II score distribution by sex is summarized in Table 2.

### 3.3. Psychometric Properties

Internal consistency was high for the HPLP-II total score (Cronbach’s alpha = 0.961; standardized alpha = 0.961) and acceptable to high across subscales (alpha range: 0.812–0.900). Corrected item-total correlations supported the coherence of the global score and subscales, with median corrected item-total correlations of 0.557 for the total scale and 0.525–0.680 across subscales. Factorability diagnostics supported factor analysis (KMO = 0.954; Bartlett’s test $\chi_{1326}^{2}=13,838.49$, p<0.001). As a descriptive eigenvalue check, PCA identified nine components with eigenvalues greater than 1; the first six components explained 52.8% of total variance. Ordinal EFA using polychoric correlations, minimum-residual extraction, and oblimin rotation was added for the four-point HPLP-II items. Parallel analysis suggested eight factors; therefore, the six-factor ordinal EFA was retained only as an exploratory comparison with the theoretical six-domain HPLP-II structure. The six-factor ordinal model had RMSR = 0.025, TLI = 0.776, RMSEA = 0.077, and a significant chi-square test. Thirty-seven items had absolute primary loadings of at least 0.40, and six items had absolute secondary loadings of at least 0.30. Full item and reliability results, ranked item means, PCA scree diagnostics, ordinal EFA diagnostics, pattern loadings, factor correlations, and cross-loading summaries are provided in Supplementary Tables S2, S3 and S6–S8 and Supplementary Figure S2. These psychometric analyses were interpreted descriptively and not as confirmatory validation evidence.

### 3.4. Empirical HPLP-II Profiles

**Figure 2:**
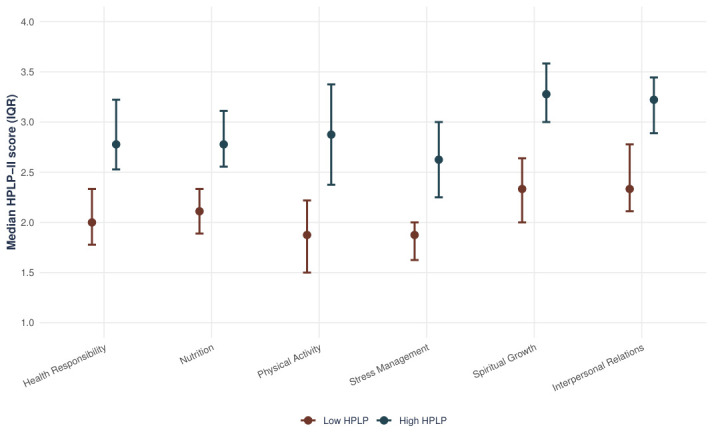
Level-based HPLP-II lifestyle profiles derived from the six subscale scores. (N=506). Clusters were derived from z-standardized subscale scores; plotted values show raw 1.00–4.00 subscale medians and interquartile ranges. The two profiles primarily reflect broadly lower versus higher HPLP-II levels across domains rather than externally validated or qualitatively distinct profile shapes.

A two-profile k-means solution based on z-standardized subscale scores was selected by average silhouette width (0.366; higher than 0.283 for the three-profile solution) and is described as a level-based profile solution. The Low HPLP profile included 294 students (58.1%), with a mean global HPLP-II score of 2.10. The High HPLP profile included 212 students (41.9%), with a mean global HPLP-II score of 2.95. The profiles differed across all six dimensions, particularly Stress Management, Physical Activity, Health Responsibility, and Spiritual Growth (Figure 2). The z-standardized centers were uniformly negative for the Low HPLP profile and uniformly positive for the High HPLP profile, indicating a broad level separation across domains rather than sharply distinct qualitative patterns. Consistent with a level rather than shape distinction, the two profile centers were almost perfectly collinear across the six z-standardized subscales (Pearson correlation of nearly −1.00) and preserved the same rank order of subscale deviations. The two-profile assignment coincided with a simple median split of the global HPLP-II score for 92.1 percent of students (Cohen’s kappa = 0.84), and a k-means solution based on principal-component scores reproduced the broad lower-versus-higher separation with moderate agreement (73.7 percent; kappa = 0.46; Supplementary Table S12). The two-profile solution therefore functions as a data-driven severity classification rather than as a set of qualitatively distinct lifestyle types.

Health-professional advice seeking was more frequent in the High HPLP profile than in the Low HPLP profile (37.3% vs. 19.0%; p<0.001, Cramer’s V=0.203). Profiles were also associated with health-information source (p=0.012), academic stress (p=0.013), vacation-period stress (p=0.007), and willingness to improve lifestyle (p<0.001). Mean academic stress was lower in the High HPLP profile than in the Low HPLP profile (7.55 vs. 7.97).

### 3.5. Primary Robust Multivariable Associations

The primary HC3 robust multivariable model explained 19.3% of the variance (adjusted $R^{2}=15.1\%$; robust Wald $F_{25,480}=5.31$, p<0.001). Regression coefficients for the main substantive model terms are shown in Table 3. The complete primary model, including all adjustment covariates and reference categories, is reported in Supplementary Table S10. Health-professional advice seeking was associated with a higher global HPLP-II score (b=0.242, 95% CI: 0.140, 0.344; p<0.001) after adjustment for sociodemographic, academic, behavioral, household, and information-source covariates.

The single-item perceived academic stress rating (b=−0.031, 95% CI: −0.061, −0.0005; p=0.047) and vacation-period stress rating (b=−0.023, 95% CI: −0.044, −0.001; p=0.036) showed small inverse adjusted associations with HPLP-II scores, with confidence intervals close to the null. Willingness to improve lifestyle was associated with higher scores (b=0.049, 95% CI: 0.020, 0.077; p<0.001). Compared with social media as the reference source of health information, research articles (b=0.243, p<0.001) and official websites (b=0.104, p=0.041) were associated with higher HPLP-II scores. Tobacco use had a negative association with a confidence interval crossing the null (b=−0.157, p=0.064), and alcohol use was not associated with the global score (p=0.489).

### 3.6. Robust and Sensitivity Analyses

Empirical lowest-quartile HPLP-II status was defined as a global score at or below the sample 25th percentile, ≤2.058, corresponding to 132 students because nine students had scores tied at the threshold value; this was a sample-specific secondary outcome, not a validated low-HPLP-II category. In the robust logistic model, health-professional advice seeking was associated with lower odds of empirical lowest-quartile HPLP-II status (OR = 0.49, 95% CI: 0.27, 0.88; p=0.017), while vacation-period stress was associated with higher odds (OR = 1.21, 95% CI: 1.08, 1.35; p<0.001). Research articles as a health-information source were also associated with lower odds of empirical lowest-quartile HPLP-II status relative to social media (OR = 0.30, 95% CI: 0.13, 0.72; p=0.007). Tobacco and alcohol use were not significantly associated with empirical lowest-quartile HPLP-II status in this model.

**Figure 3:**
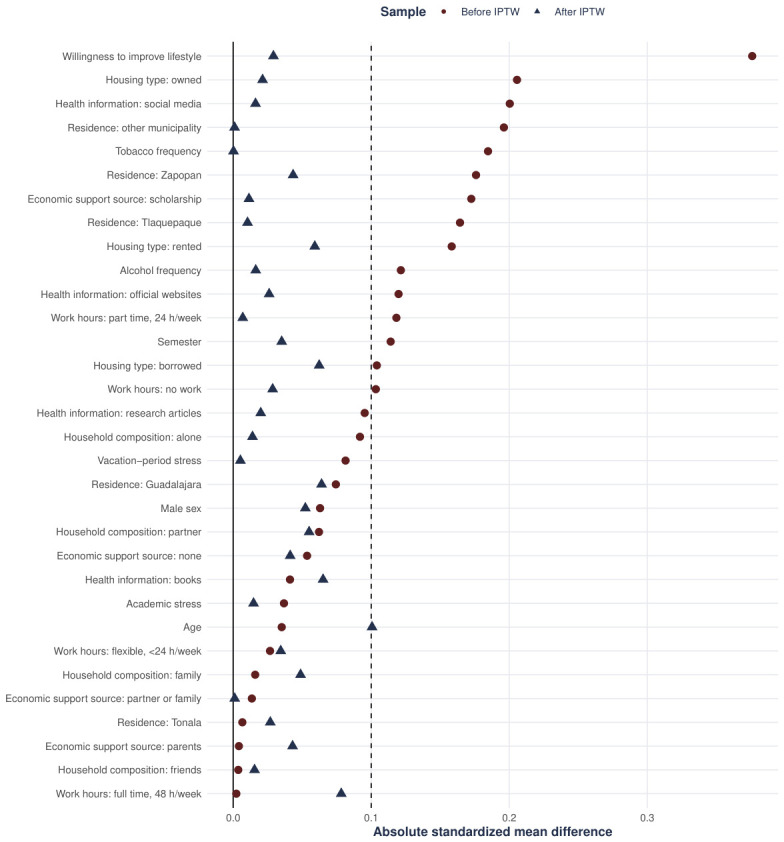
Covariate balance before and after stabilized inverse probability weighting for health-professional advice seeking. Points show absolute standardized mean differences before weighting and after truncated stabilized weighting; the dashed vertical line marks the conventional 0.10 threshold.

The IPTW sensitivity analysis reduced observed covariate imbalance for health-professional advice seeking. Across the 32 displayed covariate rows, the maximum absolute standardized mean difference decreased from 0.376 before weighting to 0.101 after truncated stabilized weighting, with one covariate remaining only marginally above the conventional 0.10 threshold (Figure 3). Propensity-score distributions suggested acceptable overlap between advice-seeking groups, although positivity and unmeasured confounding remain assumptions. The effective sample size after truncated weighting was 445.6. In the IPTW marginal weighted model, health-professional advice seeking remained associated with higher global HPLP-II scores (b=0.228, 95% CI: 0.118, 0.339; p<0.001). In the IPTW model additionally adjusted for stress and willingness to improve lifestyle, the health-professional advice association was similar (b=0.232, 95% CI: 0.126, 0.338; p<0.001).

Two additional HC3 sensitivity models addressed potential construct overlap and covariate specification. When the Health Responsibility subscale was excluded from the global HPLP-II score, health-professional advice seeking retained a positive adjusted coefficient (b=0.217, 95% CI: 0.114, 0.320; p<0.001). When willingness to improve lifestyle was removed from the primary model, the advice-seeking coefficient also remained positive (b=0.272, 95% CI: 0.169, 0.375; p<0.001). Detailed propensity-score distribution, balance diagnostics, overlap plots, subscale-specific models, item-level results, and these additional sensitivity models are provided in the Supplementary Materials.

### 3.7. Exploratory Interaction Analysis

An exploratory interaction model was fitted to examine whether the association between academic stress and global HPLP-II score differed by health-professional advice seeking status. The interaction coefficient for health-professional advice seeking by academic stress was positive but not statistically significant (b=0.043, p=0.134). This result does not provide evidence of statistical moderation at the prespecified p<0.05 threshold.

The model-based visualization is provided in Supplementary Figure S5 for hypothesis generation only. A cautious interpretation is that students who reported health-professional advice seeking had higher model-estimated mean HPLP-II scores overall, particularly across the mid-to-high academic-stress range, although the difference in slopes by advice-seeking status was statistically uncertain.

## 4. Discussion

This study was revised to emphasize one primary contribution: the adjusted cross-sectional association between health-professional advice seeking and global HPLP-II scores among undergraduate nursing students. Five findings are most relevant. First, health-professional advice seeking was associated with higher global HPLP-II scores in the primary robust model and in IPTW sensitivity analysis. Second, this association remained positive in additional models excluding the Health Responsibility subscale from the outcome and excluding willingness to improve lifestyle from the covariate set. Third, academic stress and vacation-period stress showed small inverse adjusted associations with global HPLP-II scores, whereas tobacco use had a negative association with a confidence interval crossing the null after robust adjustment. Fourth, the HPLP-II showed high internal consistency, although the ordinal exploratory factor results indicate that dimensional structure should not be treated as confirmatory evidence. Fifth, two sample-derived level-based profiles separated students with broadly lower versus higher HPLP-II scores. The exploratory interaction between academic stress and health-professional advice seeking was not statistically significant.

The descriptive HPLP-II pattern is consistent with prior research indicating that nursing students frequently report incomplete adoption of health-promoting behaviors despite their professional training in health promotion (Al-Kandari et al., 2008; Chow et al., 2018; Gurusamy et al., 2022; Hwang & Oh, 2020; Mak et al., 2018). The regional psychometric literature also supports treating Spanish-language HPLP-II applications as context-sensitive rather than automatically transportable across student populations (Enríquez Reyna et al., 2022; Pérez-Fortis et al., 2012; Zambrano Bermeo et al., 2024). The relatively higher scores in Interpersonal Relations and Spiritual Growth may reflect the importance of social ties, family support, peer connection, and meaning-making as coping resources during professional education (Chow et al., 2018; Mak et al., 2018; Slemon et al., 2021). In contrast, lower scores in Stress Management and Physical Activity are compatible with the time constraints, clinical demands, and academic pressure described in nursing education literature (Chernomas & Shapiro, 2013; Labrague, 2024; Pulido-Martos et al., 2012).

The sex-based difference in Physical Activity is noteworthy. Although the global lifestyle score and most subscales did not differ by sex, male students reported higher Physical Activity scores. This finding may reflect differences in time availability, cultural norms, safety perceptions, access to exercise facilities, domestic responsibilities, or preferences for exercise modalities. The present analysis cannot determine the source of the difference, but it may warrant attention to barriers that could differ by sex and social context when designing physical-activity promotion strategies for nursing students.

The association between health-professional advice seeking and higher HPLP-II scores was consistent in the primary robust model and in the IPTW sensitivity analysis after weighting reduced observed covariate imbalance. This finding should be interpreted carefully. HPLP-II Health Responsibility includes items related to reporting symptoms, asking health professionals questions, discussing health concerns, and seeking guidance, so the exposure and global outcome may conceptually overlap. However, the advice-seeking coefficient remained positive when the Health Responsibility subscale was excluded from the global score, suggesting that the association was not limited to those overlapping items. This sensitivity analysis reduces concern about direct item overlap but does not prove that the exposure is conceptually independent of health awareness, help-seeking orientation, or access to services. This pattern is compatible with, but does not establish, greater reported access to health-related guidance, reinforcement, or self-care resources among students who seek health-professional advice. It may also reflect unmeasured differences between students who seek advice and those who do not, including health awareness, family resources, prior health concerns, personality traits, or access to services. Propensity-score weighting helps adjust for observed covariates but does not transform this cross-sectional analysis into a causal design (Austin, 2011; Rosenbaum & Rubin, 1983). Therefore, the result supports an association between advice seeking and healthier lifestyle scores, not an estimated causal effect of professional advice. More specifically, the IPTW analysis was used only to test whether this association was robust to imbalance in measured covariates and was not intended to estimate an average treatment effect; because advice seeking and HPLP-II scores were assessed at the same cross-sectional time point, the analysis cannot establish which of the two precedes the other. In practical terms, the adjusted advice-seeking difference of 0.242 HPLP-II points corresponds to roughly 28 percent of the 0.85-point gap in mean global HPLP-II score between the lower- and higher-scoring profiles, a modest but non-trivial magnitude relative to the overall spread of lifestyle scores.

The single-item perceived academic stress rating showed an inverse association with global HPLP-II score. This aligns with literature in which stress in nursing education has been reported alongside poorer coping, burnout symptoms, reduced rest, and less consistent self-care (Gibbons et al., 2010). However, the directionality cannot be determined. Higher stress may coincide with lower engagement in healthy behaviors, but students with fewer self-care resources may also experience academic demands as more stressful. Longitudinal data would be needed to clarify temporal ordering and estimate within-person changes over time.

The exploratory moderation analysis should not be overinterpreted. Students who reported health-professional advice seeking had higher model-estimated mean HPLP-II scores overall, particularly across mid-to-high academic-stress levels, but the stress-by-advice-seeking interaction was not statistically significant. Consequently, the findings do not support a claim that the stress-HPLP-II association differs by health-professional advice-seeking status in this sample. At most, the visual pattern can inform hypotheses for future studies with longitudinal designs, clearer exposure definitions, and greater power to test interaction effects.

### 4.1. Strengths

This study has several strengths. It used a relatively large sample of undergraduate nursing students, analyzed the HPLP-II as a continuous scale, evaluated reliability and exploratory structure in the analytic sample, and used HC3 robust standard errors for the primary model. It added ordinal exploratory psychometric analyses using polychoric correlations for the four-point HPLP-II items and reported the complete primary model in the Supplementary Materials. It also used level-based profile analysis to summarize multidimensional lifestyle score patterns and IPTW as a propensity-score sensitivity analysis for health-professional advice seeking. Finally, it distinguishes primary adjusted associations from exploratory interaction analyses, reducing the risk of overstating moderation findings.

### 4.2. Limitations

The main limitation is the cross-sectional design, which precludes causal inference and assessment of temporal ordering. Participation was voluntary through an electronic questionnaire, and invitation counts and response rates by semester could not be reconstructed from the de-identified analytic file; therefore, self-selection and incomplete representativeness remain possible. Because willingness to complete a voluntary health survey may itself be related to health engagement, students who seek health-professional advice may have been more likely to participate, and such selective participation could have inflated the observed advice-seeking association. All measures were self-reported, which may introduce recall bias and social desirability bias. Perceived academic stress, vacation-period stress, and willingness to improve lifestyle were measured with single-item numerical ratings, which reduces respondent burden but limits content validity and does not capture the multidimensional structure that validated multi-item stress or motivation scales could provide. Willingness to improve lifestyle is temporally ambiguous in this design and may function as a prior motivational factor, a correlate of advice seeking, or a marker closely related to the outcome; the sensitivity model excluding this covariate partially addresses, but does not eliminate, this concern. Health-professional advice seeking was measured with a broad binary item that specified health, nutrition, stress-management, or physical-activity advice but did not include an explicit recall period. It did not capture type of professional, timing, duration, frequency, quality, or purpose of advice seeking; consequently, it may combine heterogeneous pathways such as nutrition or exercise guidance, medical advice, and psychological or therapeutic support, potentially diluting or obscuring subscale-specific associations. In addition, advice seeking may overlap conceptually with HPLP-II Health Responsibility items; the modified-score sensitivity analysis reduces this concern but cannot remove all construct-related overlap. The propensity score adjusted only for measured covariates and cannot account for unobserved confounding. The model explained a modest proportion of HPLP-II variance, indicating that other individual, institutional, and contextual factors are likely related to students’ health-promoting behaviors. The ordinal EFA remained exploratory: parallel analysis suggested eight factors, while the six-factor model was retained for theoretical comparability, and fit indices did not support confirmatory validation of the six-dimensional structure. The k-means profiles are data-driven, primarily separated lower versus higher levels across domains, and require external validation before being used for classification or screening. Because these profiles were derived from the six theoretical subscale scores, whose dimensional structure received only exploratory support, the solution is partly dependent on that structure; a supplementary re-clustering on principal-component scores reproduced the broad level separation only moderately, reinforcing that the two-profile solution is a data-driven, level-based summary rather than a confirmed typology. The empirical lowest-quartile HPLP-II outcome was sample-specific and should not be interpreted as a validated diagnostic category. The interaction analysis was exploratory and non-significant. Finally, results come from a single university setting and may not generalize to all nursing students or institutions.

### 4.3. Implications for Nursing Education

The findings are consistent with considering student wellness as part of professional formation, not only as an individual responsibility. Future work could also move beyond variable-by-variable associations and level-based profiling toward network-analytic approaches that model how stress, coping, support seeking, and well-being interrelate as a system, which may offer a more integrated account of student wellness than the two-profile summary reported here (Commodari et al., 2026; Cui et al., 2026). Institutional attention to access to mental health care, nutrition advice, physical-activity opportunities, and stress-management resources may be relevant components of institutional wellness strategies and should be evaluated prospectively. However, the present results should be used to motivate further evaluation and institutional planning, not as evidence that a specific advice or support service is effective.

## 5. Conclusions

Undergraduate nursing students in this sample reported generally moderate to low health-promoting lifestyles, with the lowest scores in Stress Management and Physical Activity. The HPLP-II showed high internal consistency, but ordinal exploratory factor analysis should be interpreted as descriptive evidence rather than confirmatory validation. Two sample-derived level-based profiles descriptively separated students with broadly lower versus higher health-promoting behaviors. Higher perceived academic and vacation-period stress ratings showed small inverse adjusted associations with global HPLP-II scores, while health-professional advice seeking showed positive adjusted associations in robust, IPTW, and additional sensitivity analyses. The exploratory interaction between academic stress and health-professional advice seeking was not statistically significant, so the study does not provide evidence that the stress-HPLP-II association differs by advice-seeking status. Future longitudinal studies are needed to evaluate whether structured institutional support precedes changes in health-promoting behaviors, and intervention studies would be required to test effectiveness.

## Figures and Tables

**Table 1:** Sociodemographic and behavioral characteristics stratified by sex (*N* = 506).

Variable	Total (*N* = 506)	Female (*n* = 403)	Male (*n* = 103)	*p* Value
Age (years) (Median, IQR)	21.0 (20.0, 22.8)	21.0 (20.0, 22.0)	21.0 (20.0, 23.0)	0.067
Employment Status				
Study only	262 (51.8)	219 (54.3)	43 (41.7)	0.030
Study and work	244 (48.2)	184 (45.7)	60 (58.3)	
Working Hours per Week				
No work	262 (51.8)	219 (54.3)	43 (41.7)	0.012
Flexible shift (<24 h)	109 (21.5)	88 (21.8)	21 (20.4)	
Part-time (24 h)	71 (14.0)	54 (13.4)	17 (16.5)	
Full-time (48 h)	64 (12.6)	42 (10.4)	22 (21.4)	
Economic Support				
Yes	390 (77.1)	324 (80.4)	66 (64.1)	<0.001
No	116 (22.9)	79 (19.6)	37 (35.9)	
Current Residence				
Guadalajara	189 (37.4)	146 (36.2)	43 (41.7)	0.128
Zapopan	132 (26.1)	103 (25.6)	29 (28.2)	
San Pedro Tlaquepaque	73 (14.4)	63 (15.6)	10 (9.7)	
Tonalá	57 (11.3)	42 (10.4)	15 (14.6)	
Other municipalities	55 (10.9)	49 (12.2)	6 (5.8)	
Health-Professional Advice Seeking				
Yes	135 (26.7)	110 (27.3)	25 (24.3)	0.621
No	371 (73.3)	293 (72.7)	78 (75.7)	
Tobacco Smoking				
Yes	44 (8.7)	32 (7.9)	12 (11.7)	0.319
No	462 (91.3)	371 (92.1)	91 (88.3)	
Alcohol Consumption				
Yes	318 (62.8)	245 (60.8)	73 (70.9)	0.076
No	188 (37.2)	158 (39.2)	30 (29.1)	

**Table 2:** Descriptive statistics and sex-based comparison of HPLP-II scores (range 1.00–4.00).

HPLP-II Dimension	Total (*N* = 506)	Female (*n* = 403)	Male (*n* = 103)	*p* Value
Global HPLP-II Score (Median, IQR)	2.40 (2.06, 2.79)	2.40 (2.06, 2.79)	2.46 (2.09, 2.79)	0.448
Interpersonal Relations (Median, IQR)	2.78 (2.33, 3.11)	2.78 (2.33, 3.17)	2.67 (2.22, 3.06)	0.157
Spiritual Growth (Median, IQR)	2.67 (2.22, 3.22)	2.67 (2.22, 3.22)	2.67 (2.17, 3.22)	0.772
Nutrition (Median, IQR)	2.33 (2.00, 2.78)	2.33 (2.00, 2.78)	2.44 (2.11, 2.78)	0.261
Health Responsibility (Median, IQR)	2.33 (1.89, 2.67)	2.33 (1.89, 2.67)	2.33 (2.00, 2.67)	0.766
Physical Activity (Median, IQR)	2.12 (1.75, 2.88)	2.00 (1.62, 2.81)	2.50 (2.00, 3.06)	<0.001
Stress Management (Median, IQR)	2.12 (1.75, 2.50)	2.12 (1.75, 2.50)	2.12 (1.88, 2.62)	0.082

**Table 3:** Selected unstandardized coefficients from the primary multivariable HC3 robust linear model for global HPLP-II score. The complete primary model is provided in Supplementary Table S10.

Model Term	Unstandardized Coefficient (*b*)	Robust SE	95% CI	*p* Value
Health-professional advice seeking (yes vs. no)	0.242	0.052	[0.140, 0.344]	<0.001
Academic stress rating (0–10)	−0.031	0.016	[−0.061, −0.0005]	0.047
Vacation-period stress (0–10)	−0.023	0.011	[−0.044, −0.001]	0.036
Willingness to improve lifestyle (0–10)	0.049	0.014	[0.020, 0.077]	<0.001
Flexible work (<24 h/week)	0.148	0.059	[0.033, 0.263]	0.012
Official websites as health-information source	0.104	0.051	[0.005, 0.204]	0.041
Research articles as health-information source	0.243	0.068	[0.108, 0.377]	<0.001
Tobacco use	−0.157	0.084	[−0.323, 0.009]	0.064
Alcohol use	0.032	0.047	[−0.059, 0.124]	0.489

Note: Coefficients are unstandardized adjusted mean differences in global HPLP-II score units. Full model also adjusted for age, sex, semester, working-hours categories, economic support, residence, housing type, household composition, and all health-information-source categories. Reference categories are no health-professional advice seeking, female sex, no work, Guadalajara residence, owned housing, family household composition, social media as health-information source, no tobacco use, and no alcohol use.

## Data Availability

The de-identified analytic dataset, R analysis code, documentation, and supporting reproducibility files are publicly available in Zenodo at https://doi.org/10.5281/zenodo.20382383. Raw identifiable or potentially identifiable data are not publicly available due to participant-confidentiality and institutional restrictions.

## Supplementary Materials

The following supporting information can be downloaded at: https://www.mdpi.com/article/10.3390/ejihpe16070101/s1, Figure S1: Propensity score overlap by health-professional advice seeking status. Dashed vertical lines mark group medians; Figure S2: PCA scree plot for HPLP-II items. The dashed horizontal line marks eigenvalue = 1; PCA was used as a descriptive eigenvalue check rather than as the primary dimensional model; Figure S3: HPLP-II item mean scores ranked from lowest to highest. The dashed vertical line marks the global HPLP-II mean; Figure S4: Subscale-specific coefficients for health-professional advice seeking and academic stress from HC3 robust models. Points show unstandardized coefficients and whiskers show 95% confidence intervals; Figure S5: Model-estimated mean global HPLP-II scores by academic stress and health-professional advice seeking status in the exploratory interaction model. This visualization is provided for hypothesis generation only and should not be interpreted as evidence of statistical moderation; Table S1: Descriptive summary of analytic covariates used in the primary and propensity-score models; Table S2: Variable recoding, analytic mapping, and HPLP-II item allocation; Table S3: Internal consistency reliability of the HPLP-II total scale and subscales; Table S4: HC3 robust subscale models for each HPLP-II dimension; Table S5: Propensity score model diagnostics and covariate balance; Table S6: HPLP-II items ranked by mean score, from lowest to highest; Table S7: Ordinal exploratory factor-analysis diagnostics for HPLP-II items; Table S8: Ordinal EFA pattern loadings based on polychoric correlations; Table S9: K-means profile-selection diagnostics and z-standardized cluster centers; Table S10: Complete primary multivariable HC3 robust linear model for global HPLP-II score; Table S11: Additional HC3 sensitivity analyses for health-professional advice seeking; Table S12: Robustness of the two-profile solution: level-versus-shape check, concordance with a global-score split, and re-clustering on principal-component scores.

## Abbreviations

The following abbreviations are used in this manuscript:

CUCS	Centro Universitario de Ciencias de la Salud
EFA	Exploratory factor analysis
HC3	Heteroskedasticity-consistent standard errors, type 3
HPLP-II	Health-Promoting Lifestyle Profile II
IPTW	Inverse probability of treatment weighting
IQR	Interquartile range
KMO	Kaiser–Meyer–Olkin
PCA	Principal component analysis
