# Supplementary material for: Health-Promoting Lifestyle Scores, Academic Stress, and Health-Professional Advice Seeking Among Undergraduate Nursing Students: A Cross-Sectional Study
Source: Eur J Investig Health Psychol Educ. 2026 Jul 15;16(7):101. doi: 10.3390/ejihpe16070101 (PMC13407694; doi:10.3390/ejihpe16070101)
Supplement: Supplementary file 1 [file ejihpe-16-00101-s001.zip › supplementary_material.pdf]

Supplementary Materials

Health-Promoting Lifestyle Scores, Academic Stress, and Health-Professional Advice Seeking among Undergraduate Nursing Students: A Cross-Sectional Study

Alexis Emmanuel Salinas-Santoyo[1], Gabriela Luna-Hernández[2], Victor Horacio Orozco-Covarrubias[3]
Janvier Andre Martinez-Godinez[1], Jaime Briseno-Ramírez[4,5,*], Cecilia Alejandra Zamora-Figueroa[1,*]

[1]Departamento de Enfermería para la Atención, Desarrollo y Preservación de la Salud Comunitaria, Especialidad de Enfermería en Salud Pública, Centro Universitario de Ciencias de la Salud, Universidad de Guadalajara, Guadalajara, Jalisco 44340, Mexico.
[2]Departamento de Alimentación y Nutrición, Centro Universitario de Ciencias de la Salud, Universidad de Guadalajara, Guadalajara, Jalisco 44340, Mexico.
[3]Departamento de Salud-Enfermedad como Proceso Individual y Colectivo, Centro Universitario de Tlajomulco, Universidad de Guadalajara, Tlajomulco de Zúñiga, Jalisco 45641, Mexico.
[4]División Salud, Centro Universitario de Tlajomulco, Universidad de Guadalajara, Tlajomulco de Zúñiga, Jalisco 45641, Mexico.
[5]Hospital Civil de Oriente, Tonalá, Jalisco 45425, Mexico.

*Corresponding authors: jaime.briseno@academicos.udg.mx; cecilia.zamora@academicos.udg.mx

Supplementary Methods

This file documents the complete analytic covariate summary, analytic recoding, HPLP-II psychometric reliability, ordinal exploratory factor and profile diagnostics, HC3 robust subscale-specific outcome models, the complete primary HC3 robust model, propensity score diagnostics, additional sensitivity analyses, two-profile robustness diagnostics, item-level HPLP-II rankings, and exploratory interaction visualization used to support the main manuscript.

Supplementary Tables

Table S1. Descriptive summary of analytic covariates used in the primary and propensity-score models.

Covariate	Analytic role	Overall summary (N = 506)
Health-professional advice seeking	Primary exposure / PS treatment	Yes: 135 (26.7%); no: 371 (73.3%)
Age	Outcome model / PS covariate	21.0 (20.0, 22.8)
Sex	Outcome model / PS covariate	Male: 103 (20.4%); female: 403 (79.6%)
Academic semester	Outcome model / PS covariate	5.0 (3.0, 7.0)
Weekly working hours	Outcome model / PS covariate	No work: 262 (51.8%); Flexible <24 h/week: 109 (21.5%); Part-time 24 h/week: 71 (14.0%); Full-time 48 h/week: 64 (12.6%)
Economic support	Outcome model covariate	Yes: 390 (77.1%); no: 116 (22.9%)
Economic-support source	PS covariate	None: 117 (23.1%); Parents: 368 (72.7%); Scholarship: 9 (1.8%); Partner/family: 12 (2.4%); Other: 0 (0.0%)[a]

Continued on next page

Table S1. Descriptive summary of analytic covariates used in the primary and propensity-score models (continued).

Covariate	Analytic role	Overall summary (N = 506)
Residence	Outcome model / PS covariate	Guadalajara: 189 (37.4%); Zapopan: 132 (26.1%); San Pedro Tlaquepaque: 73 (14.4%); Tonalá: 57 (11.3%); Other municipality: 55 (10.9%)
Housing type	Outcome model / PS covariate	Owned: 320 (63.2%); Rented: 146 (28.9%); Borrowed: 40 (7.9%)
Household composition	Outcome model / PS covariate	Family: 459 (90.7%); Alone: 13 (2.6%); Friends: 19 (3.8%); Partner: 15 (3.0%)
Health-information source	Outcome model / PS covariate	Social media: 243 (48.0%); Official websites: 170 (33.6%); Research articles: 77 (15.2%); Books: 16 (3.2%); Other: 0 (0.0%)
Academic-period stress	Outcome model / PS covariate	8.0 (7.0, 9.0)
Vacation-period stress	Outcome model / PS covariate	5.0 (3.0, 7.0)
Willingness to improve lifestyle	Outcome model / PS covariate / sensitivity covariate	9.0 (8.0, 10.0)
Tobacco smoking	Outcome model covariate	Yes: 44 (8.7%); no: 462 (91.3%)
Tobacco-use frequency	PS covariate	0.0 (0.0, 0.0)
Alcohol consumption	Outcome model covariate	Yes: 318 (62.8%); no: 188 (37.2%)
Alcohol-use frequency	PS covariate	1.0 (0.0, 1.0)

Note: Values are n (%) for categorical variables and median (IQR) for continuous or ordinal variables. This table summarizes the 506 complete analytic records used in the primary multivariable model and the propensity-score balance set. PS = propensity score; HPLP-II = Health-Promoting Lifestyle Profile II. [a]One participant reported receiving economic support but selected no source of support; source categories are shown as reported.

Table S2. Variable recoding, analytic mapping, and HPLP-II item allocation.

Panel	Analytic variable	Brief source description	Coding / derivation	Role / construct
A. Baseline and analytic covariates	Edad	Age in completed years	Continuous, years	Outcome model covariate / PS model
	Sexo_Coded	Sex assigned at birth	Male = 1; female = 0	Outcome model covariate / PS model
	Semestre_Correcto	Academic semester, repaired from the HPLP-II sheet	Numeric academic semester; aligned to HPLP-II responses	Outcome model covariate / PS model
	Trabaja	Employment status	Study and work = 1; study only = 0	Descriptive covariate
	Horas_Trabajo_Cat	Weekly working hours	No work, flexible < 24 h/week, part-time 24 h/week, full-time 48 h/week	Outcome model covariate / PS model
	Apoyo_Coded	Economic support	Yes = 1; no = 0	Outcome model covariate
	Apoyo_Origen_Cat	Main source of economic support	None, parents, scholarship, partner or family	PS model covariate
	Residencia_Cat	Current residence	Guadalajara, Zapopan, Tlaquepaque, Tonalá, other municipality	Outcome model covariate / PS model
	Tipo_Vivienda_Cat	Housing type	Owned, rented, borrowed	Outcome model covariate / PS model
	Composicion_ Hogar_Cat	Household composition	Family, alone, friends, partner	Outcome model covariate / PS model
	Info_Salud_Cat	Main source of health information	Social media, official websites, research articles, books, other	Outcome model covariate / PS model
	Asesoria_Coded	Health-professional advice seeking	Yes = 1; no = 0	Primary exposure
	Academic_Stress	Academic-period stress	Continuous 0–10 scale	Main predictor / outcome model
	Vacation_Stress	Vacation-period stress	Continuous 0–10 scale	Main predictor / outcome model
	Fuma	Tobacco consumption	Yes = 1; no = 0	Outcome model covariate
	Toma_Alcohol	Alcohol consumption	Yes = 1; no = 0	Outcome model covariate

Continued on next page

Table S2. Variable recoding, analytic mapping, and HPLP-II item allocation (continued).

Panel	Analytic variable	Brief source description	Coding / derivation	Role / construct
	Tabaco_Freq_Ordinal	Tobacco-use frequency	Ordinal frequency score from original response options	PS model covariate
	Alcohol_Freq_Ordinal	Alcohol-use frequency	Ordinal frequency score from original response options	PS model covariate
	Disposicion_Mejorar	Willingness to improve lifestyle	Continuous 0–10 scale	Outcome model covariate / PS model / sensitivity covariate
	propensity_score	Derived from logistic PS model	Predicted probability of health-professional advice seeking	IPTW construction / diagnostic
B. HPLP-II scale scores	HPLP_Total_Mean	All 52 HPLP-II items	Mean of items recoded to 1–4	Primary outcome
	HPLP_No_HR_Mean	HPLP-II excluding Health Responsibility	Weighted mean of Nutrition, Physical Activity, Stress Management, Spiritual Growth, and Interpersonal Relations subscales using their item counts (43 items)	Sensitivity outcome
	Sub_Responsabilidad_Salud	HPLP-II items 3, 9, 15, 21, 27, 33, 39, 45, 51	Mean of listed items recoded to 1–4	Health Responsibility subscale outcome
	Sub_Nutricion	HPLP-II items 2, 8, 14, 20, 26, 32, 38, 44, 50	Mean of listed items recoded to 1–4	Nutrition subscale outcome
	Sub_Actividad_Fisica	HPLP-II items 4, 10, 16, 22, 28, 34, 40, 46	Mean of listed items recoded to 1–4	Physical Activity subscale outcome
	Sub_Manejo_Estres	HPLP-II items 5, 11, 17, 23, 29, 35, 41, 47	Mean of listed items recoded to 1–4	Stress Management subscale outcome
	Sub_Crecimiento_Espiritual	HPLP-II items 6, 12, 18, 24, 30, 36, 42, 48, 52	Mean of listed items recoded to 1–4	Spiritual Growth subscale outcome
	Sub_Relaciones_Interpersonales	HPLP-II items 1, 7, 13, 19, 25, 31, 37, 43, 49	Mean of listed items recoded to 1–4	Interpersonal Relations subscale outcome
C. HPLP-II item-level map	HPLP01	Discusses problems and concerns with close people	Original 0–3 response recoded by adding 1; final range 1–4	Interpersonal Relations
	HPLP02	Chooses a diet low in fat, saturated fat, and cholesterol	Original 0–3 response recoded by adding 1; final range 1–4	Nutrition
	HPLP03	Reports unusual signs or symptoms to a doctor or health professional	Original 0–3 response recoded by adding 1; final range 1–4	Health Responsibility
	HPLP04	Follows a planned exercise program	Original 0–3 response recoded by adding 1; final range 1–4	Physical Activity
	HPLP05	Gets enough sleep	Original 0–3 response recoded by adding 1; final range 1–4	Stress Management
	HPLP06	Feels that personal growth and positive change are occurring	Original 0–3 response recoded by adding 1; final range 1–4	Spiritual Growth
	HPLP07	Praises other people for their achievements	Original 0–3 response recoded by adding 1; final range 1–4	Interpersonal Relations
	HPLP08	Limits sugar and sugar-containing foods	Original 0–3 response recoded by adding 1; final range 1–4	Nutrition
	HPLP09	Reads or watches programs about improving health	Original 0–3 response recoded by adding 1; final range 1–4	Health Responsibility
	HPLP10	Performs vigorous exercise for at least 20 minutes three or more times per week	Original 0–3 response recoded by adding 1; final range 1–4	Physical Activity
	HPLP11	Takes time to relax every day	Original 0–3 response recoded by adding 1; final range 1–4	Stress Management
	HPLP12	Believes life has purpose	Original 0–3 response recoded by adding 1; final range 1–4	Spiritual Growth
	HPLP13	Maintains meaningful and enriching relationships	Original 0–3 response recoded by adding 1; final range 1–4	Interpersonal Relations
	HPLP14	Eats 6–11 servings of bread, cereals, rice, or pasta each day	Original 0–3 response recoded by adding 1; final range 1–4	Nutrition
	HPLP15	Asks health professionals questions to understand instructions	Original 0–3 response recoded by adding 1; final range 1–4	Health Responsibility
	HPLP16	Performs light-to-moderate physical activity for 30–40 minutes, five or more times per week	Original 0–3 response recoded by adding 1; final range 1–4	Physical Activity
	HPLP17	Accepts things in life that cannot be changed	Original 0–3 response recoded by adding 1; final range 1–4	Stress Management

Continued on next page

Table S2. Variable recoding, analytic mapping, and HPLP-II item allocation (continued).

Panel	Analytic variable	Brief source description	Coding / derivation	Role / construct
	HPLP18	Looks forward to the future	Original 0–3 response recoded by adding 1; final range 1–4	Spiritual Growth
	HPLP19	Spends time with close friends	Original 0–3 response recoded by adding 1; final range 1–4	Interpersonal Relations
	HPLP20	Eats 2–4 servings of fruit every day	Original 0–3 response recoded by adding 1; final range 1–4	Nutrition
	HPLP21	Seeks a second opinion when health recommendations are in doubt	Original 0–3 response recoded by adding 1; final range 1–4	Health Responsibility
	HPLP22	Participates in recreational physical activities such as swimming, dancing, or cycling	Original 0–3 response recoded by adding 1; final range 1–4	Physical Activity
	HPLP23	Focuses on pleasant thoughts at bedtime	Original 0–3 response recoded by adding 1; final range 1–4	Stress Management
	HPLP24	Feels satisfied and at peace with oneself	Original 0–3 response recoded by adding 1; final range 1–4	Spiritual Growth
	HPLP25	Finds it easy to show concern, love, and affection to others	Original 0–3 response recoded by adding 1; final range 1–4	Interpersonal Relations
	HPLP26	Eats 3–5 servings of vegetables every day	Original 0–3 response recoded by adding 1; final range 1–4	Nutrition
	HPLP27	Discusses health concerns with health professionals	Original 0–3 response recoded by adding 1; final range 1–4	Health Responsibility
	HPLP28	Performs stretching exercises at least three times per week	Original 0–3 response recoded by adding 1; final range 1–4	Physical Activity
	HPLP29	Uses specific methods to control stress	Original 0–3 response recoded by adding 1; final range 1–4	Stress Management
	HPLP30	Works toward long-term goals in life	Original 0–3 response recoded by adding 1; final range 1–4	Spiritual Growth
	HPLP31	Has affectionate physical contact with important people	Original 0–3 response recoded by adding 1; final range 1–4	Interpersonal Relations
	HPLP32	Eats 2–3 servings of milk, yogurt, or cheese each day	Original 0–3 response recoded by adding 1; final range 1–4	Nutrition
	HPLP33	Checks the body monthly for physical changes or warning signs	Original 0–3 response recoded by adding 1; final range 1–4	Health Responsibility
	HPLP34	Gets exercise during usual daily activities such as walking, stairs, or parking farther away	Original 0–3 response recoded by adding 1; final range 1–4	Physical Activity
	HPLP35	Balances time between work and leisure	Original 0–3 response recoded by adding 1; final range 1–4	Stress Management
	HPLP36	Finds each day interesting and challenging	Original 0–3 response recoded by adding 1; final range 1–4	Spiritual Growth
	HPLP37	Seeks ways to meet intimacy needs	Original 0–3 response recoded by adding 1; final range 1–4	Interpersonal Relations
	HPLP38	Eats 2–3 servings of meat, poultry, fish, beans, eggs, or nuts each day	Original 0–3 response recoded by adding 1; final range 1–4	Nutrition
	HPLP39	Asks health professionals how to take good care of oneself	Original 0–3 response recoded by adding 1; final range 1–4	Health Responsibility
	HPLP40	Checks pulse when exercising	Original 0–3 response recoded by adding 1; final range 1–4	Physical Activity
	HPLP41	Practices relaxation or meditation for 15–20 minutes daily	Original 0–3 response recoded by adding 1; final range 1–4	Stress Management
	HPLP42	Is aware of what is important in life	Original 0–3 response recoded by adding 1; final range 1–4	Spiritual Growth
	HPLP43	Seeks support from people who care	Original 0–3 response recoded by adding 1; final range 1–4	Interpersonal Relations
	HPLP44	Reads nutrition labels for fat and sodium content in packaged foods	Original 0–3 response recoded by adding 1; final range 1–4	Nutrition
	HPLP45	Attends educational programs about personal health care	Original 0–3 response recoded by adding 1; final range 1–4	Health Responsibility
	HPLP46	Reaches target heart rate when exercising	Original 0–3 response recoded by adding 1; final range 1–4	Physical Activity

Continued on next page

Table S2. Variable recoding, analytic mapping, and HPLP-II item allocation (continued).

Panel	Analytic variable	Brief source description	Coding / derivation	Role / construct
	HPLP47	Maintains balance to prevent fatigue	Original 0–3 response recoded by adding 1; final range 1–4	Stress Management
	HPLP48	Feels connected with a force greater than oneself	Original 0–3 response recoded by adding 1; final range 1–4	Spiritual Growth
	HPLP49	Resolves differences with others through dialogue and compromise	Original 0–3 response recoded by adding 1; final range 1–4	Interpersonal Relations
	HPLP50	Eats breakfast	Original 0–3 response recoded by adding 1; final range 1–4	Nutrition
	HPLP51	Seeks guidance or advice when needed	Original 0–3 response recoded by adding 1; final range 1–4	Health Responsibility
	HPLP52	Tries new experiences and challenges	Original 0–3 response recoded by adding 1; final range 1–4	Spiritual Growth

Table S3. Internal consistency reliability of the HPLP-II total scale and subscales.

Scale	Items	Mean	SD	Cronbach α	Std. α	Item-rest r range
Global HPLP-II	52	2.46	0.52	0.961	0.961	0.36–0.69
Health Responsibility	9	2.34	0.61	0.857	0.856	0.44–0.72
Nutrition	9	2.44	0.55	0.812	0.814	0.34–0.64
Physical Activity	8	2.28	0.76	0.886	0.886	0.50–0.77
Stress Management	8	2.18	0.58	0.842	0.841	0.43–0.69
Spiritual Growth	9	2.72	0.66	0.900	0.901	0.58–0.75
Interpersonal Relations	9	2.73	0.59	0.840	0.839	0.46–0.66

Note: Scores are means on the 1–4 HPLP-II response scale. Corrected item-rest correlations correlate each item with the sum of the remaining items in the same scale.

Table S4. HC3 robust subscale models for each HPLP-II dimension.

Outcome	Predictor	b	Robust SE	95% CI	p	FDR q	Adj. R^2
Health responsibility	Health-professional advice seeking: yes vs no	0.361	0.061	[0.242, 0.481]	< 0.001	< 0.001	0.146
	Academic stress, academic period	-0.029	0.018	[-0.064, 0.005]	0.099	0.197	
	Stress, vacation period	-0.009	0.013	[-0.034, 0.016]	0.468	0.562	
	Willingness to improve lifestyle	0.038	0.017	[0.005, 0.070]	0.023	0.027	
	Tobacco use	-0.086	0.100	[-0.282, 0.110]	0.391	0.391	
	Alcohol use	0.038	0.056	[-0.072, 0.149]	0.494	0.958	
Nutrition	Health-professional advice seeking: yes vs no	0.249	0.057	[0.138, 0.361]	< 0.001	< 0.001	0.140
	Academic stress, academic period	-0.023	0.016	[-0.055, 0.010]	0.169	0.254	
	Stress, vacation period	-0.016	0.011	[-0.039, 0.006]	0.152	0.304	
	Willingness to improve lifestyle	0.045	0.016	[0.015, 0.076]	0.004	0.008	
	Tobacco use	-0.204	0.085	[-0.372, -0.037]	0.017	0.101	
	Alcohol use	0.005	0.050	[-0.093, 0.102]	0.926	0.958	
Physical activity	Health-professional advice seeking: yes vs no	0.373	0.075	[0.225, 0.521]	< 0.001	< 0.001	0.129
	Academic stress, academic period	-0.013	0.022	[-0.055, 0.030]	0.556	0.586	
	Stress, vacation period	-0.008	0.015	[-0.037, 0.021]	0.582	0.582	
	Willingness to improve lifestyle	0.071	0.020	[0.032, 0.110]	< 0.001	0.002	
	Tobacco use	-0.183	0.097	[-0.373, 0.007]	0.060	0.126	

Continued on next page

Table S4. HC3 robust subscale models for each HPLP-II dimension (continued).

Outcome	Predictor	b	Robust SE	95% CI	p	FDR q	Adj. R^2
	Alcohol use	0.076	0.071	[-0.063, 0.215]	0.285	0.855	
Stress management	Health-professional advice seeking: yes vs no	0.156	0.059	[0.039, 0.272]	0.009	0.011	0.114
	Academic stress, academic period	-0.071	0.018	[-0.107, -0.036]	< 0.001	< 0.001	
	Stress, vacation period	-0.011	0.012	[-0.035, 0.013]	0.374	0.562	
	Willingness to improve lifestyle	0.029	0.017	[-0.004, 0.062]	0.086	0.086	
	Tobacco use	-0.087	0.092	[-0.269, 0.094]	0.345	0.391	
	Alcohol use	-0.003	0.053	[-0.107, 0.102]	0.958	0.958	
Spiritual growth	Health-professional advice seeking: yes vs no	0.177	0.067	[0.046, 0.309]	0.008	0.011	0.127
	Academic stress, academic period	-0.043	0.019	[-0.080, -0.006]	0.023	0.070	
	Stress, vacation period	-0.050	0.013	[-0.076, -0.024]	< 0.001	0.001	
	Willingness to improve lifestyle	0.067	0.020	[0.028, 0.105]	< 0.001	0.002	
	Tobacco use	-0.199	0.107	[-0.410, 0.011]	0.063	0.126	
	Alcohol use	0.005	0.061	[-0.115, 0.126]	0.930	0.958	
Interpersonal relations	Health-professional advice seeking: yes vs no	0.142	0.061	[0.021, 0.262]	0.021	0.021	0.076
	Academic stress, academic period	-0.009	0.017	[-0.043, 0.025]	0.586	0.586	
	Stress, vacation period	-0.038	0.012	[-0.062, -0.014]	0.002	0.006	
	Willingness to improve lifestyle	0.043	0.017	[0.009, 0.077]	0.013	0.020	
	Tobacco use	-0.176	0.105	[-0.382, 0.031]	0.095	0.143	
	Alcohol use	0.073	0.056	[-0.036, 0.183]	0.189	0.855	

Note: Each outcome was modeled separately as a continuous subscale mean on the 1–4 HPLP-II scale. Models used the same covariate structure as the primary HC3 robust model. FDR q values are Benjamini–Hochberg adjusted within each predictor term across the six subscales.

Table S5. Propensity score model diagnostics and covariate balance.

Panel A. Propensity score distribution by health-professional advice seeking status

Group	N	Mean	SD	Median	IQR	Range
Advice seeking: yes	135	0.296	0.085	0.302	0.234–0.351	0.069–0.530
Advice seeking: no	371	0.256	0.085	0.255	0.204–0.312	0.046–0.539

Panel B. Covariate balance before and after truncated stabilized inverse probability weighting

Covariate	Type	SMD	Weighted SMD	|Weighted SMD|	Pre	Post
Willingness to improve lifestyle	Continuous	0.376	-0.029	0.029	> 0.10	Balanced
Housing type: owned	Binary	0.206	0.021	0.021	> 0.10	Balanced
Health information: social media	Binary	-0.200	0.016	0.016	> 0.10	Balanced
Residence: other municipality	Binary	-0.196	0.001	0.001	> 0.10	Balanced
Tobacco frequency	Continuous	0.185	0.000	0.000	> 0.10	Balanced
Residence: Zapopan	Binary	0.176	-0.043	0.043	> 0.10	Balanced
Economic support source: scholarship	Binary	0.173	-0.011	0.011	> 0.10	Balanced
Residence: Tlaquepaque	Binary	-0.164	-0.010	0.010	> 0.10	Balanced
Housing type: rented	Binary	-0.158	-0.059	0.059	> 0.10	Balanced
Alcohol frequency	Continuous	0.121	0.016	0.016	> 0.10	Balanced
Health information: official websites	Binary	0.120	-0.026	0.026	> 0.10	Balanced
Work hours: part time, 24 h/week	Binary	-0.118	-0.007	0.007	> 0.10	Balanced
Semester	Continuous	0.114	-0.035	0.035	> 0.10	Balanced
Housing type: borrowed	Binary	-0.104	0.062	0.062	> 0.10	Balanced
Work hours: no work	Binary	0.103	0.029	0.029	> 0.10	Balanced
Health information: research articles	Binary	0.095	-0.020	0.020	Balanced	Balanced

Continued on next page

Table S5. Propensity score model diagnostics and covariate balance (continued).

Covariate	Type	SMD	Weighted SMD	|Weighted SMD|	Pre	Post
Household composition: alone	Binary	0.092	-0.014	0.014	Balanced	Balanced
Vacation-period stress	Continuous	0.081	0.005	0.005	Balanced	Balanced
Residence: Guadalajara	Binary	0.074	0.064	0.064	Balanced	Balanced
Male sex	Binary	-0.063	0.052	0.052	Balanced	Balanced
Household composition: partner	Binary	-0.062	-0.055	0.055	Balanced	Balanced
Economic support source: none	Binary	-0.054	-0.041	0.041	Balanced	Balanced
Health information: books	Binary	0.041	0.065	0.065	Balanced	Balanced
Academic stress	Continuous	0.037	0.015	0.015	Balanced	Balanced
Age	Continuous	0.035	-0.101	0.101	Balanced	Marginal > 0.10
Work hours: flexible, < 24 h/week	Binary	-0.027	0.034	0.034	Balanced	Balanced
Household composition: family	Binary	-0.016	0.049	0.049	Balanced	Balanced
Economic support source: partner or family	Binary	-0.014	-0.001	0.001	Balanced	Balanced
Residence: Tonalá	Binary	-0.007	-0.027	0.027	Balanced	Balanced
Economic support source: parents	Binary	-0.004	0.043	0.043	Balanced	Balanced
Household composition: friends	Binary	-0.004	-0.015	0.015	Balanced	Balanced
Work hours: full time, 48 h/week	Binary	-0.002	-0.078	0.078	Balanced	Balanced

Note: Positive standardized mean differences (SMDs) indicate higher values among students who sought health-professional advice. The weighted SMD column is included as a balance diagnostic for the truncated stabilized inverse probability weighting sensitivity analysis. The maximum absolute SMD decreased from 0.376 before weighting to 0.101 after weighting; one covariate row remained marginally above the conventional 0.10 threshold.

Table S6. HPLP-II items ranked by mean score, from lowest to highest.

Rank	Item	Subscale	Brief item description	Mean	SD	Median	IQR
1	HPLP41	Stress Management	Practices relaxation or meditation for 15–20 minutes daily	1.75	0.89	2.0	1.0–2.0
2	HPLP45	Health Responsibility	Attends educational programs about personal health care	1.76	0.81	2.0	1.0–2.0
3	HPLP29	Stress Management	Uses specific methods to control stress	1.89	0.90	2.0	1.0–2.0
4	HPLP04	Physical Activity	Follows a planned exercise program	2.00	1.03	2.0	1.0–3.0
5	HPLP09	Health Responsibility	Reads or watches programs about improving health	2.02	0.83	2.0	1.0–2.0
6	HPLP47	Stress Management	Maintains balance to prevent fatigue	2.06	0.82	2.0	2.0–2.0
7	HPLP46	Physical Activity	Reaches target heart rate when exercising	2.06	0.99	2.0	1.0–3.0
8	HPLP14	Nutrition	Eats 6–11 servings of bread, cereals, rice, or pasta each day	2.09	0.83	2.0	2.0–3.0
9	HPLP22	Physical Activity	Participates in recreational physical activities such as swimming, dancing, or cycling	2.12	0.96	2.0	1.0–3.0
10	HPLP40	Physical Activity	Checks pulse when exercising	2.14	1.01	2.0	1.0–3.0
11	HPLP44	Nutrition	Reads nutrition labels for fat and sodium content in packaged foods	2.16	0.99	2.0	1.0–3.0
12	HPLP05	Stress Management	Gets enough sleep	2.16	0.74	2.0	2.0–3.0
13	HPLP02	Nutrition	Chooses a diet low in fat, saturated fat, and cholesterol	2.22	0.81	2.0	2.0–3.0
14	HPLP11	Stress Management	Takes time to relax every day	2.24	0.88	2.0	2.0–3.0
15	HPLP08	Nutrition	Limits sugar and sugar-containing foods	2.29	0.79	2.0	2.0–3.0
16	HPLP39	Health Responsibility	Asks health professionals how to take good care of oneself	2.31	0.91	2.0	2.0–3.0
17	HPLP27	Health Responsibility	Discusses health concerns with health professionals	2.31	0.90	2.0	2.0–3.0
18	HPLP35	Stress Management	Balances time between work and leisure	2.32	0.85	2.0	2.0–3.0
19	HPLP28	Physical Activity	Performs stretching exercises at least three times per week	2.32	1.04	2.0	2.0–3.0
20	HPLP03	Health Responsibility	Reports unusual signs or symptoms to a doctor or health professional	2.33	0.93	2.0	2.0–3.0
21	HPLP01	Interpersonal Relations	Discusses problems and concerns with close people	2.36	0.82	2.0	2.0–3.0
22	HPLP48	Spiritual Growth	Feels connected with a force greater than oneself	2.37	0.99	2.0	2.0–3.0
23	HPLP37	Interpersonal Relations	Seeks ways to meet intimacy needs	2.37	0.90	2.0	2.0–3.0
24	HPLP23	Stress Management	Focuses on pleasant thoughts at bedtime	2.40	0.86	2.0	2.0–3.0

Continued on next page

Table S6. HPLP-II items ranked by mean score, from lowest to highest (continued).

Rank	Item	Subscale	Brief item description	Mean	SD	Median	IQR
25	HPLP10	Physical Activity	Performs vigorous exercise for at least 20 minutes three or more times per week	2.40	1.09	2.0	2.0–3.0
26	HPLP36	Spiritual Growth	Finds each day interesting and challenging	2.41	0.83	2.0	2.0–3.0
27	HPLP21	Health Responsibility	Seeks a second opinion when health recommendations are in doubt	2.42	0.87	2.0	2.0–3.0
28	HPLP32	Nutrition	Eats 2–3 servings of milk, yogurt, or cheese each day	2.47	0.89	2.0	2.0–3.0
29	HPLP20	Nutrition	Eats 2–4 servings of fruit every day	2.48	0.85	2.0	2.0–3.0
30	HPLP26	Nutrition	Eats 3–5 servings of vegetables every day	2.50	0.85	2.0	2.0–3.0
31	HPLP16	Physical Activity	Performs light-to-moderate physical activity for 30–40 minutes, five or more times per week	2.54	1.00	2.0	2.0–3.0
32	HPLP43	Interpersonal Relations	Seeks support from people who care	2.55	1.00	2.0	2.0–3.0
33	HPLP15	Health Responsibility	Asks health professionals questions to understand instructions	2.55	0.92	3.0	2.0–3.0
34	HPLP06	Spiritual Growth	Feels that personal growth and positive change are occurring	2.57	0.83	3.0	2.0–3.0
35	HPLP24	Spiritual Growth	Feels satisfied and at peace with oneself	2.57	0.87	2.0	2.0–3.0
36	HPLP34	Physical Activity	Gets exercise during usual daily activities such as walking, stairs, or parking farther away	2.63	1.02	3.0	2.0–4.0
37	HPLP17	Stress Management	Accepts things in life that cannot be changed	2.65	0.85	3.0	2.0–3.0
38	HPLP33	Health Responsibility	Checks the body monthly for physical changes or warning signs	2.67	0.91	3.0	2.0–3.0
39	HPLP52	Spiritual Growth	Tries new experiences and challenges	2.70	0.91	3.0	2.0–3.0
40	HPLP19	Interpersonal Relations	Spends time with close friends	2.71	0.84	3.0	2.0–3.0
41	HPLP51	Health Responsibility	Seeks guidance or advice when needed	2.72	0.95	3.0	2.0–4.0
42	HPLP38	Nutrition	Eats 2–3 servings of meat, poultry, fish, beans, eggs, or nuts each day	2.74	0.84	3.0	2.0–3.0
43	HPLP25	Interpersonal Relations	Finds it easy to show concern, love, and affection to others	2.80	0.96	3.0	2.0–4.0
44	HPLP49	Interpersonal Relations	Resolves differences with others through dialogue and compromise	2.80	0.89	3.0	2.0–3.0
45	HPLP31	Interpersonal Relations	Has affectionate physical contact with important people	2.81	0.93	3.0	2.0–4.0
46	HPLP30	Spiritual Growth	Works toward long-term goals in life	2.89	0.90	3.0	2.0–4.0
47	HPLP12	Spiritual Growth	Believes life has purpose	2.93	0.91	3.0	2.0–4.0
48	HPLP50	Nutrition	Eats breakfast	2.98	0.94	3.0	2.0–4.0
49	HPLP13	Interpersonal Relations	Maintains meaningful and enriching relationships	3.00	0.83	3.0	2.0–4.0
50	HPLP18	Spiritual Growth	Looks forward to the future	3.03	0.84	3.0	2.0–4.0
51	HPLP42	Spiritual Growth	Is aware of what is important in life	3.03	0.90	3.0	2.0–4.0
52	HPLP07	Interpersonal Relations	Praises other people for their achievements	3.22	0.80	3.0	3.0–4.0

Note: Item responses were recoded to the standard 1–4 HPLP-II metric before ranking. Item descriptions are brief English paraphrases of the administered Spanish HPLP-II items. Lower means indicate behaviors reported less frequently and may represent priority targets for intervention.

Table S7. Ordinal exploratory factor-analysis diagnostics for HPLP-II items.

Diagnostic	Value
Correlation matrix	Polychoric
Extraction / rotation	Minimum residual / Oblimin
Requested factors	6
Parallel-analysis suggested factors	8
Complete observations	506
RMSR	0.025
TLI	0.776
RMSEA	0.077
BIC	-2318.2
Model chi-square	4088.9
df	1029
P value	< 0.001

Loading summary	Value
Items with primary loading greater than or equal to 0.30	49
Items with primary loading greater than or equal to 0.40	37
Items with secondary loading greater than or equal to 0.30	6
Median absolute primary loading	0.503

Factor	OF1	OF2	OF3	OF4	OF5	OF6
OF1	1.00	0.38	0.48	0.48	0.48	0.21
OF2	0.38	1.00	0.45	0.51	0.17	0.33
OF3	0.48	0.45	1.00	0.48	0.37	0.25
OF4	0.48	0.51	0.48	1.00	0.34	0.26
OF5	0.48	0.17	0.37	0.34	1.00	0.05
OF6	0.21	0.33	0.25	0.26	0.05	1.00

Note: Analyses used polychoric correlations for four-point HPLP-II items, minimum-residual extraction, and oblimin rotation. Parallel analysis suggested eight factors; the six-factor model was retained as an exploratory structure aligned with the six theoretical HPLP-II domains, not as confirmatory validation evidence. OF = ordinal factor; RMSR = root mean square residual; TLI = Tucker–Lewis index; RMSEA = root mean square error of approximation.

Table S8. Ordinal EFA pattern loadings based on polychoric correlations.

Item	Theoretical domain	OF1	OF2	OF3	OF4	OF5	OF6
Item_01	Interpersonal Relations	0.16	0.11	0.12	-0.05	0.37	0.06
Item_02	Nutrition	0.22	0.38	0.34	0.11	-0.27	-0.05
Item_03	Health Responsibility	0.07	-0.03	0.52	0.09	0.07	0.11
Item_04	Physical Activity	-0.05	0.81	0.07	0.04	-0.05	0.01
Item_05	Stress Management	0.42	0.11	-0.20	0.12	-0.07	0.21
Item_06	Spiritual Growth	0.54	0.15	0.08	0.09	-0.00	0.00
Item_07	Interpersonal Relations	0.17	-0.02	0.11	0.21	0.33	-0.25
Item_08	Nutrition	0.24	0.25	0.28	0.21	-0.33	-0.06
Item_09	Health Responsibility	0.12	0.19	0.28	0.09	-0.16	0.18

Continued on next page

Table S8. Ordinal EFA pattern loadings based on polychoric correlations (continued).

Item	Theoretical domain	OF1	OF2	OF3	OF4	OF5	OF6
Item_10	Physical Activity	-0.01	1.01	-0.05	-0.05	-0.04	-0.05
Item_11	Stress Management	0.42	0.16	-0.09	0.04	0.08	0.26
Item_12	Spiritual Growth	0.78	-0.02	0.07	0.02	0.04	-0.05
Item_13	Interpersonal Relations	0.30	0.03	0.02	0.22	0.38	-0.12
Item_14	Nutrition	-0.12	-0.01	-0.00	0.36	0.18	0.24
Item_15	Health Responsibility	0.07	0.13	0.51	0.03	0.09	-0.02
Item_16	Physical Activity	0.04	0.73	0.05	0.04	0.11	-0.07
Item_17	Stress Management	0.54	-0.04	0.05	0.08	0.08	0.01
Item_18	Spiritual Growth	0.74	0.07	-0.03	-0.01	0.09	-0.17
Item_19	Interpersonal Relations	0.23	0.06	-0.08	0.10	0.48	0.04
Item_20	Nutrition	0.16	0.13	0.00	0.58	-0.12	-0.01
Item_21	Health Responsibility	0.04	0.07	0.39	0.22	0.08	0.18
Item_22	Physical Activity	-0.01	0.60	-0.03	0.15	0.05	0.19
Item_23	Stress Management	0.68	-0.01	0.00	0.07	-0.03	0.21
Item_24	Spiritual Growth	0.83	-0.07	-0.03	0.05	0.08	0.13
Item_25	Interpersonal Relations	0.25	0.04	0.01	0.05	0.44	0.03
Item_26	Nutrition	0.04	0.08	0.11	0.69	-0.02	-0.01
Item_27	Health Responsibility	-0.07	0.00	0.59	0.27	0.07	0.16
Item_28	Physical Activity	-0.07	0.78	-0.07	0.13	0.11	0.11
Item_29	Stress Management	0.12	0.12	0.13	0.14	0.07	0.56
Item_30	Spiritual Growth	0.48	0.10	0.22	0.03	0.24	-0.14
Item_31	Interpersonal Relations	0.10	0.10	0.01	0.18	0.58	-0.03
Item_32	Nutrition	-0.09	0.04	-0.03	0.64	0.19	0.05
Item_33	Health Responsibility	0.16	0.03	0.25	0.21	0.21	0.03
Item_34	Physical Activity	0.01	0.38	0.21	0.18	0.13	-0.01
Item_35	Stress Management	0.46	0.11	-0.01	0.21	0.06	0.26
Item_36	Spiritual Growth	0.63	-0.02	0.06	0.12	0.03	0.19
Item_37	Interpersonal Relations	0.11	0.01	0.16	0.22	0.24	0.17
Item_38	Nutrition	0.08	0.02	0.15	0.53	0.08	-0.17
Item_39	Health Responsibility	-0.02	0.04	0.60	0.18	0.05	0.17
Item_40	Physical Activity	0.12	0.33	0.17	-0.10	0.09	0.33
Item_41	Stress Management	0.21	0.13	0.04	0.11	-0.04	0.57
Item_42	Spiritual Growth	0.58	0.01	0.25	-0.01	0.18	-0.24
Item_43	Interpersonal Relations	0.08	0.09	0.16	0.02	0.61	0.09
Item_44	Nutrition	0.07	0.15	0.43	0.06	-0.08	0.16
Item_45	Health Responsibility	-0.05	0.05	0.30	-0.02	0.07	0.62
Item_46	Physical Activity	0.04	0.48	0.21	-0.12	0.07	0.34
Item_47	Stress Management	0.38	0.11	0.06	0.07	-0.09	0.50
Item_48	Spiritual Growth	0.44	0.06	0.14	-0.05	0.15	0.23
Item_49	Interpersonal Relations	0.22	0.00	0.42	-0.03	0.28	-0.10
Item_50	Nutrition	0.36	0.24	0.11	0.15	0.08	-0.24
Item_51	Health Responsibility	0.23	0.09	0.35	0.07	0.31	-0.02
Item_52	Spiritual Growth	0.26	0.06	0.35	0.02	0.29	0.02

Note: Values are oblimin-rotated pattern coefficients from a six-factor ordinal EFA using a polychoric correlation matrix and minimum-residual extraction. Cross-loadings should be interpreted descriptively because the analysis is exploratory and the parallel analysis suggested more than six factors.

Table S9. K-means profile-selection diagnostics and z-standardized cluster centers.

Candidate profiles	Total within-cluster SS	Between-cluster SS	Average silhouette
2	1658.8	1371.2	0.366
3	1273.6	1756.4	0.283
4	1125.5	1904.5	0.223
5	1029.6	2000.4	0.218

Profile	HR	Nutrition	PA	Stress	Spiritual	Interpersonal
Low HPLP	-0.58	-0.56	-0.53	-0.58	-0.62	-0.55
High HPLP	0.81	0.77	0.73	0.81	0.86	0.76

Note: Clustering used z-standardized HPLP-II subscale scores with 100 random starts. The two-profile solution had the highest average silhouette width. HR = Health Responsibility; PA = Physical Activity.

Table S10. Complete primary multivariable HC3 robust linear model for global HPLP-II score.

Model term	*b*	Robust SE	95% CI	P value
Health-professional advice seeking: yes vs no	0.242	0.052	[0.140, 0.344]	< 0.001
Academic stress, academic period	-0.031	0.016	[-0.061, -0.000]	0.047
Stress, vacation period	-0.023	0.011	[-0.044, -0.001]	0.036
Willingness to improve lifestyle	0.049	0.014	[0.020, 0.077]	< 0.001
Age	-0.007	0.013	[-0.032, 0.017]	0.562
Male sex	0.022	0.062	[-0.101, 0.144]	0.725
Semester	-0.005	0.013	[-0.030, 0.020]	0.694
Work hours: flexible under 24 h/week	0.148	0.059	[0.033, 0.263]	0.012
Work hours: part-time 24 h/week	0.049	0.078	[-0.103, 0.202]	0.528
Work hours: full-time 48 h/week	-0.023	0.090	[-0.201, 0.154]	0.797
Economic support	-0.002	0.072	[-0.143, 0.140]	0.982
Residence: Zapopan	0.023	0.055	[-0.085, 0.132]	0.675
Residence: Tlaquepaque	0.095	0.075	[-0.052, 0.242]	0.204
Residence: Tonala	0.109	0.077	[-0.041, 0.260]	0.154
Residence: Other municipality	-0.029	0.081	[-0.187, 0.129]	0.720
Housing type: Rented	-0.076	0.061	[-0.197, 0.044]	0.213
Housing type: Borrowed	-0.131	0.068	[-0.265, 0.004]	0.057
Household composition: Alone	-0.039	0.178	[-0.388, 0.311]	0.828
Household composition: Friends	-0.019	0.111	[-0.238, 0.200]	0.864
Household composition: Partner	0.063	0.144	[-0.220, 0.347]	0.660
Health information source: Official websites	0.104	0.051	[0.005, 0.204]	0.041
Health information source: Research articles	0.243	0.068	[0.108, 0.377]	< 0.001
Health information source: Books	0.103	0.167	[-0.224, 0.430]	0.536
Tobacco use	-0.157	0.084	[-0.322, 0.009]	0.064
Alcohol use	0.032	0.047	[-0.059, 0.124]	0.489

Note: Coefficients are unstandardized adjusted mean differences in global HPLP-II score units. Reference categories are: no health-professional advice seeking, female sex, no work, Guadalajara residence, owned housing, family household composition, social media as health-information source, no tobacco use, and no alcohol use.

Table S11. Additional HC3 sensitivity analyses for health-professional advice seeking.

Sensitivity model	b	Robust SE	95% CI	P value
Modified HPLP-II excluding Health Responsibility	0.217	0.052	[0.114, 0.320]	< 0.001
Primary HPLP-II excluding willingness	0.272	0.052	[0.169, 0.375]	< 0.001

Note: Both models used HC3 robust standard errors and the same adjustment structure as the primary model, except where noted. The modified HPLP-II score excluded the Health Responsibility subscale and averaged the remaining 43 HPLP-II items using their subscale item counts.

Table S12. Robustness of the two-profile solution: level-versus-shape check, concordance with a global-score split, and re-clustering on principal-component scores.

Diagnostic	Value
Correlation between the two profiles' z-standardized subscale centers (Pearson r)	-1.00
Rank order of subscale deviations (magnitude), Low vs. High (Spearman ρ)	1.00
Low HPLP profile: all six subscale centers negative	Yes
High HPLP profile: all six subscale centers positive	Yes
Agreement of two-profile solution with a global HPLP-II median split	92.1%
Cohen's κ vs. median split	0.84
PCA-based re-clustering (first six components; 52.8% of item variance)	Low $n = 309$, High $n = 197$
Average silhouette width, PCA-based two-cluster solution	0.139
Agreement of PCA-based with subscale-based profiles	73.7%
Cohen's κ, PCA-based vs. subscale-based	0.46

Note: The two profile centers were almost perfectly collinear (constant ratio ≈ -1.39 across subscales) and preserved the same rank order of subscale deviations, indicating that the profiles differed in overall level rather than in shape. Agreement with a simple median split of the global HPLP-II score was high, confirming that the two-profile solution largely reproduces a data-driven severity split. Re-clustering on principal-component scores derived from the 52 items (rather than on the theoretical subscales) reproduced the broad lower-versus-higher separation with moderate agreement, indicating that the level-based solution is not an artifact of the theoretical subscale structure but remains sensitive to the choice of input dimensions. All analyses used z-standardized inputs with 100 random starts.

Supplementary Figures

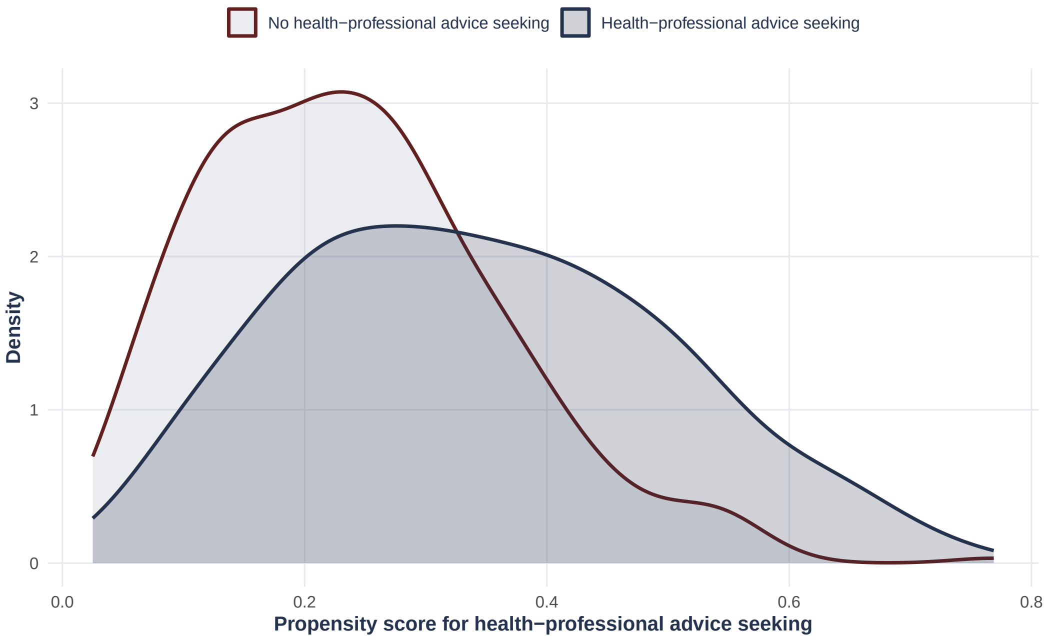

Figure S1. Propensity score overlap by health-professional advice seeking status. Dashed vertical lines mark group medians.

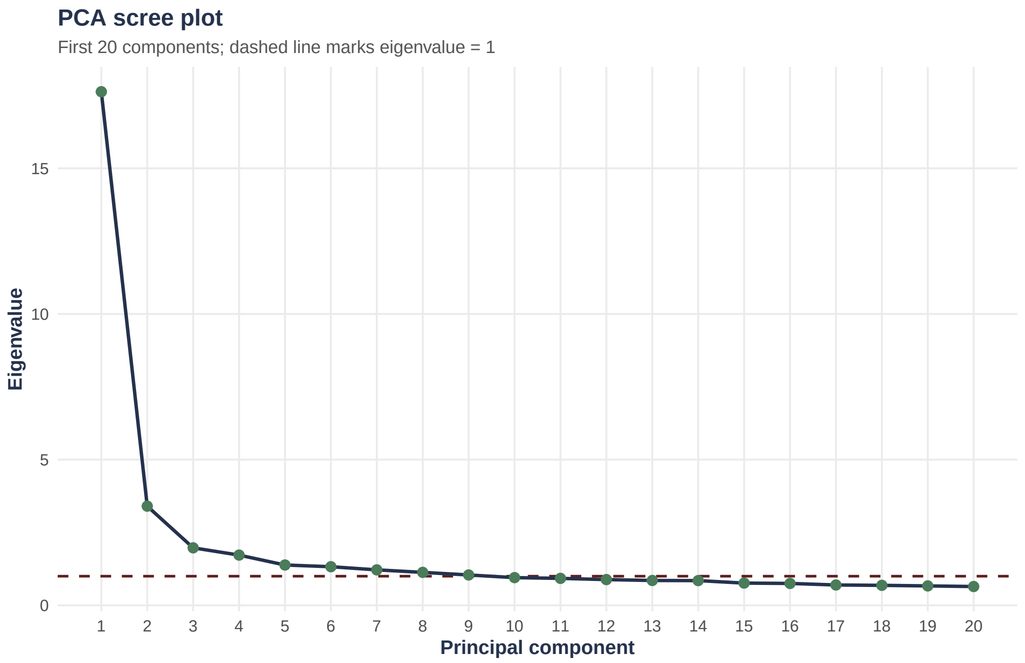

Figure S2. PCA scree plot for HPLP-II items. The dashed horizontal line marks eigenvalue = 1; PCA was used as a descriptive eigenvalue check rather than as the primary dimensional model.

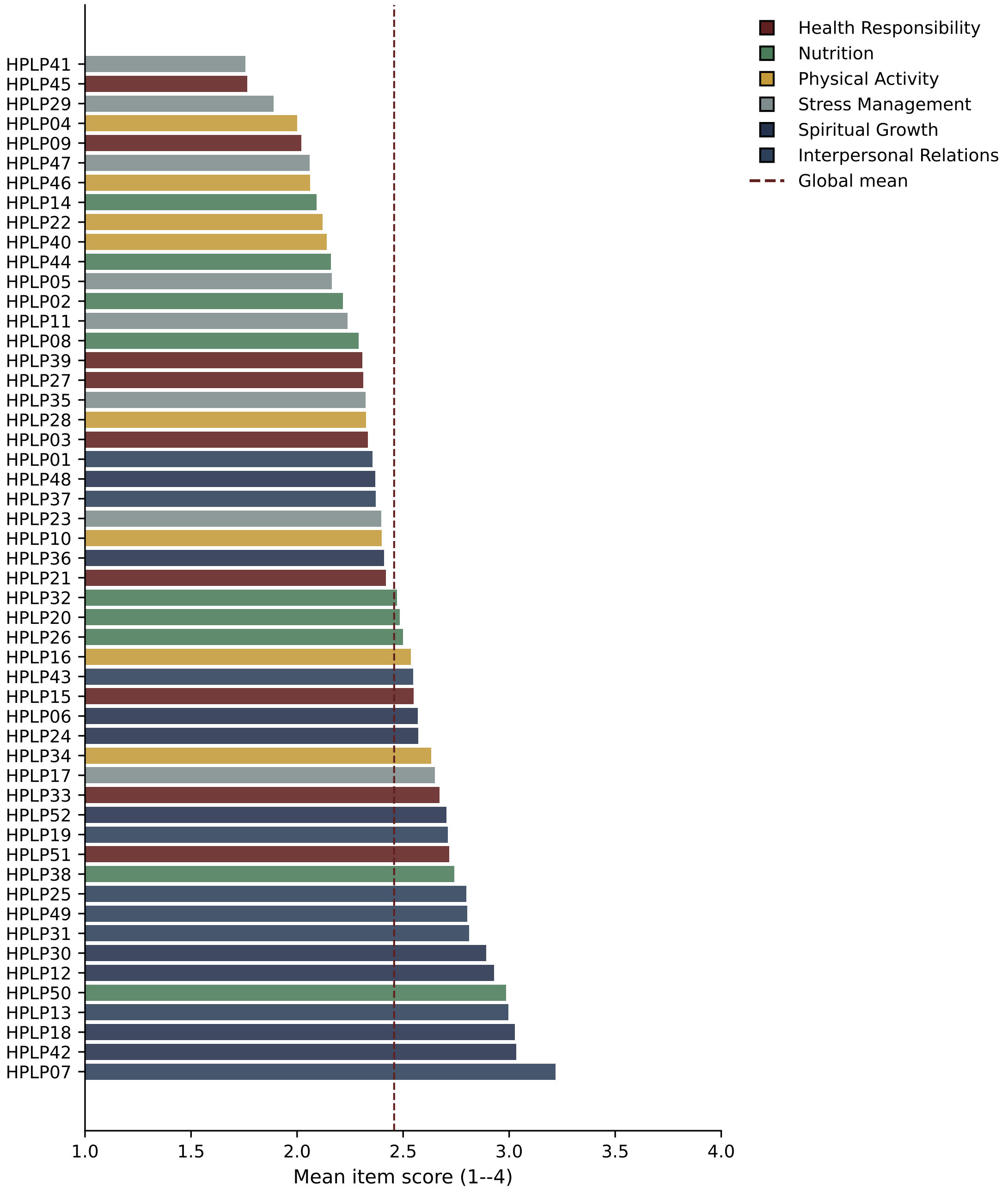

Figure S3. HPLP-II item mean scores ranked from lowest to highest. The dashed vertical line marks the global HPLP-II mean.

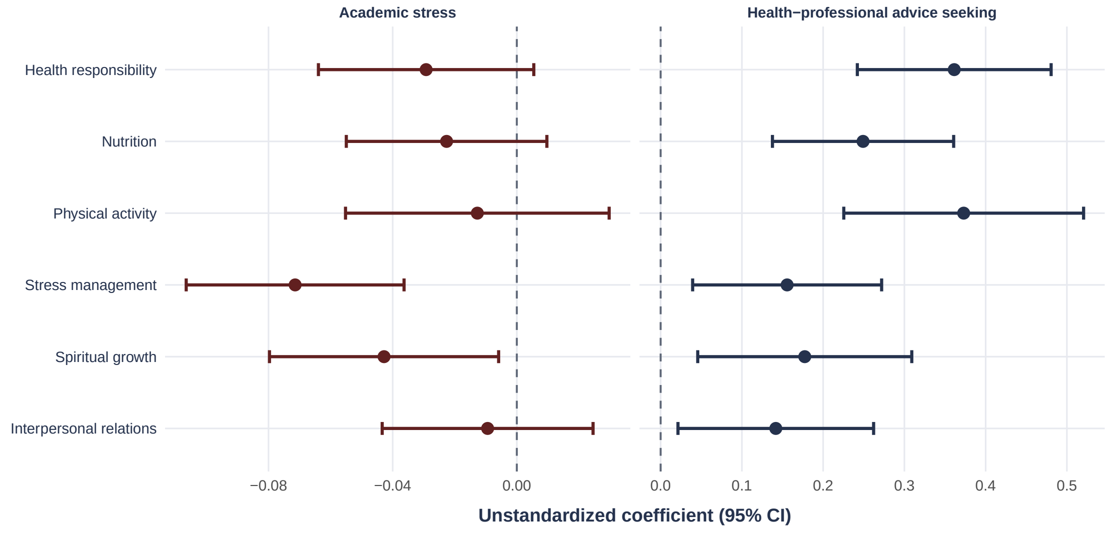

Figure S4. Subscale-specific coefficients for health-professional advice seeking and academic stress from HC3 robust models. Points show unstandardized coefficients and whiskers show 95% confidence intervals.

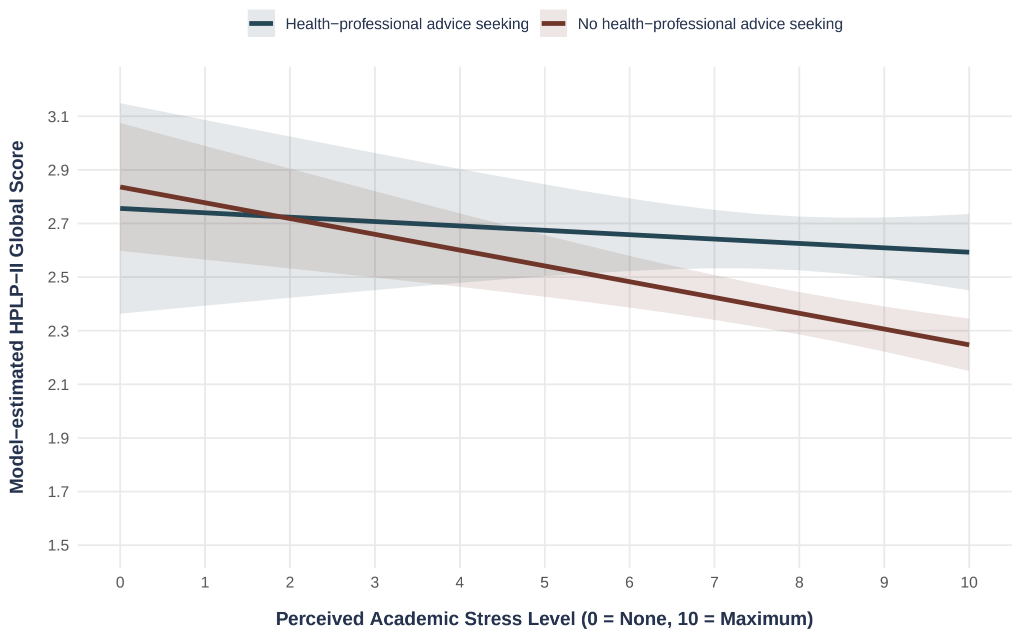

Figure S5. Model-estimated mean global HPLP-II scores by academic stress and health-professional advice seeking status in the exploratory interaction model. This visualization is provided for hypothesis generation only and should not be interpreted as evidence of statistical moderation.
